# Cost-effectiveness analysis of a prognostic risk assessment for early-stage 1-3b diabetic kidney disease patients in the United States

**DOI:** 10.1186/s12962-026-00775-4

**Published:** 2026-06-24

**Authors:** Jacie T. Cooper, John E. Schneider, Thomas Mclain, Steven Coca, Michael J. Donovan

**Affiliations:** 1 Avalon Health Economics, Miami, FL USA; 2 Renalytix, New York, NY USA; 3 Renalytix, Mount Sinai, New York, USA; 4 Ichan School of Medicine at Mt. Sinai, New York, NY USA

**Keywords:** Diabetic kidney disease, Sodium-glucose co-transporter-2 inhibitors, Chronic kidney disease, Biomarker, Kidney function, Cost effectiveness

## Abstract

**Background:**

Diabetic kidney disease (DKD) accounts for 44% of new chronic kidney disease cases and is associated with significant morbidity and mortality. Although sodium-glucose co-transporter-2 inhibitors (SGLT2is) can reduce cardiorenal outcomes, current DKD staging relying on eGFR and albuminuria (KDIGO risk categories) leaves SGLT2is underutilized in clinical practice. A new biomarker-enriched, AI-enabled risk score (KidneyIntelX™; Renalytix, Inc.) was developed to predict a progressive decline in kidney function in patients with early-stage DKD. KidneyIntelX categorizes patients as low, intermediate, or high risk for disease progression, which can guide resource utilization, prescribing of drugs such as SGLT2is, and improvements in efficiency of care. We report a cost-effectiveness analysis comparing DKD patient stratification with KidneyIntelX to KDIGO by generating an incremental cost effectiveness ratio (ICER).

**Methods:**

The model adopted a U.S. Medicare perspective and consisted of patients with DKD in stages G1-3b using KidneyIntelX or KDIGO. A 10-state Markov state transition structure was employed over a lifetime horizon which includes DKD stages 1–5, dialysis, kidney transplant, cardiovascular (CV) death, and non-CV death. Transition probabilities and risk group distributions for KidneyIntelX and KDIGO were sourced from a KidneyIntelX validation study. Evaluation resulting from KidneyIntelX or KDIGO informed SGLT2i use in the model. Cost inputs included testing, medications, and office visit costs, as well as annual costs for each DKD stage, dialysis, and kidney transplant. Quality of life for each disease state was captured as utility values informed by literature.

**Results:**

The modeled use of SGLT2i directed by KidneyIntelX led to a reduction in kidney disease progression (ESKD) and CV events, as well as dialysis starts, dialysis crashes, and kidney transplants compared to KDIGO-guided treatment. KidneyIntelX patients also spent less time in DKD stages 4 and 5 and more time in 1 through 3b. KidneyIntelX led to cost savings of about $514 per patient and quality-adjusted life year (QALY) gains of 0.028, resulting in a negative ICER. Combining SGLT2i with MRA’s further increased per patient cost savings to $530 for Medicare participants.

**Conclusions:**

Wide deployment of KidneyIntelX in Medicare patients with DKD G1-3b is expected to be cost-effective compared to KDIGO from the Medicare perspective, with a negative, dominant ICER.

**Supplementary Information:**

The online version contains supplementary material available at https://doi.org/10.1186/s12962-026-00775-4.

## Background

Chronic kidney disease (CKD) is a costly and growing epidemic in the United States (US) with greater than 37 million adults experiencing adverse consequences such as kidney failure, cardiovascular disease, and premature death [1, 2]. Once CKD progresses to advanced stages, waste products and toxins are not properly filtered leading to many of the complications of kidney disease and a diminished quality of life [2]. According to the US Centers for Disease Control and Prevention (CDC), up to 90% of adults with CKD are unaware they have the disease and of those with severe CKD, it is estimated that 40% do not know they have it [3]. The economic burden of CKD is substantial, and increases sharply as the disease progresses [4, 5]. In addition, kidney disease is a costly public health issue, contributing to a significant portion of US Medicare spending totaling $153 billion, more than 20% of the Medicare budget [6–8]. Diabetes accounts for approximately half of the cases of CKD patients in the United States [9]. Diabetic kidney disease (DKD), also known as diabetic nephropathy, affects approximately 16 million adults in the US and there are as many as 40% of individuals with type 2 diabetes (T2D) who suffer from DKD, which has adverse effects on their health-related quality of life [10]. DKD is also often linked to heart failure, as reduced kidney function increases the risk of cardiovascular (CV) related events [11]. In 2017, DKD spending accounted for 14% of all Medicare spending, totaling more than $40 billion [12]. 

CKD staging typically relies on a matrix of estimated glomerular filtration rate (eGFR) and albuminuria, per the Kidney Disease Improving Global Outcomes (KDIGO) guidelines. More than 90% of patients with eGFR and urine albumin-creatinine ratio (UACR) available through passive or active testing are in early CKD stages (stages 1–3). Therapeutic inertia at these early stages, however, is common, due to several factors: (a) only 50% of patients with diabetes receive annual UACR testing; (b) even recently validated equations for eGFR misclassify individuals as having or not having CKD in approximately 30% of cases; (c) UACR (and eGFR, to a lesser extent) are highly variable within short periods of time within the same individual (thereby confounding staging, and ability to determine progression, regression, or stability of CKD); and (d) this staging approach does not adequately risk stratify individuals for future risk of progression of disease over time, of which 20–30% will experience within 5 years [13]. 

In the past decade several new CKD therapies have been developed, including sodium-glucose co-transporter-2 inhibitors (SGLT2i), GLP-1 receptor agonists, and non-steroidal mineralocorticoid antagonists, which has led to significant advancements in the treatment of CKD and DKD. SGLT2i therapy has been shown to decrease the composite risk of dialysis, transplantation, and death due to kidney and cardiac disease by 33% in patients with T2D [14]. Despite the evidence that these agents can markedly slow progression, uptake in clinical practice remains low, with only 6–18% of eligible patients receiving them [15–17]. 

Recently, a new biomarker-enriched, machine-learning prognostic assay with a risk score (KidneyIntelX™; Renalytix) was developed and validated to predict a progressive decline in kidney function in patients with early-stage DKD [13]. KidneyIntelX yields a simple and actionable, patient-specific risk score, identifying adult with T2D who are at low, intermediate, or high risk for a progressive decline in kidney function, essentially “ruling in” patients who are at highest risk of progression and “ruling out” those who are not [18]. The development of a patient-level risk for progression tool should guide resource utilization, new drug prescriptions such as SGLT2i’s, and improve efficiency of care among physicians. A KidneyIntelX validation study demonstrated that 69% of patients scored as high risk actually experienced progression of kidney disease, compared to 40% based on KDIGO high-risk criteria, which has been considered the standard of care [13]. Conversely, only 10% of those scored as low risk by KidneyIntelX experienced progression (i.e., negative predictive value of 90%). KidneyIntelX was granted DeNovo marketing authorization in June of 2023, which marks it as a first-in-class prognostic platform to guide DKD care management [19].

KidneyIntelX can generate significant cost savings for payers through improved care management at earlier stages of CKD. A budget impact model (BIM) developed by Datar et al. (2021) quantified KidneyIntelX economic benefits of risk assessment and intervention in 100,000 early-stage DKD patients over five years [10]. The total budget impact of both KidneyIntelX and standard of care were calculated to determine potential savings. Results showed that a single, one-time KidneyIntelX test had a net savings of $10,522 per patient tested. Assuming 100% compliance with preventative measures, the net savings to the modeled health plan would reach $1.249 billion, and with 50% compliance, the net savings would be $757 million. With savings exceeding $513 million in the fifth year, each consecutive year after the breakeven point was shown to have substantial savings [10]. However, BIMs do not reflect impact of an intervention on patient lifespan or health-related quality of life (HRQoL), which are addressed in cost-effectiveness analyses (CEA). In this manuscript, we examine the incremental cost-effectiveness of KidneyIntelX compared to risk stratification using eGFR and UACR (KDIGO guidelines). The cost-effectiveness model adopts a lifetime horizon and is from a public and private payer perspective.

## Methods

### Model overview

The cost-effectiveness model estimates the cost-effectiveness of KidneyIntelX, a prognostic test for assessment of chronic (diabetic) kidney disease progression (stages 1-3b) vs. population based eGFR and UACR levels, which are currently considered the standard of care (SOC). The model is from the US public payer perspective (Medicare), with a scenario analysis from the perspective of US private payers. Transition probabilities and risk group distributions for KidneyIntelX were estimated based on cohort data from a validation study [13]. Clinical expertise was provided by a practicing nephrologist who validated model assumptions, CKD clinical pathways, and input parameters.

Patients evaluated with KidneyIntelX receive a risk score which predicts a composite kidney endpoint of progressive decline in kidney function (PDKF), which is defined as (1) an eGFR decline of ≥ 5 ml/min/1.73m^2^/year; (2) sustained decline in eGFR of ≥ 40%; or (3) one of the following: (a) kidney failure, defined as a sustained eGFR < 15 ml/min/1.73m^2^; (b) long-term maintenance dialysis; or (c) kidney transplant [10, 13]. Based on the risk score, patients are classified into one of three resulting risk groups, each of which is associated with optimal care pathways: [10]

1. **High risk**: (Score > 85) Patients should be considered for the most intensive therapy and/or referral to a nephrologist combined with aggressive medical management.
2. **Intermediate risk**: (Score 50–85), which represents the population-level risk in the validation study. Patients should be more frequently monitored (e.g., at least twice annually) by PCPs or endocrinologists with administration of guideline-directed therapies.
3. **Low risk**: (Score < 50) Patients should remain on their current treatment regimen with behavior reinforcement and should be monitored by their PCP or endocrinologist annually.

**Fig. 1:**
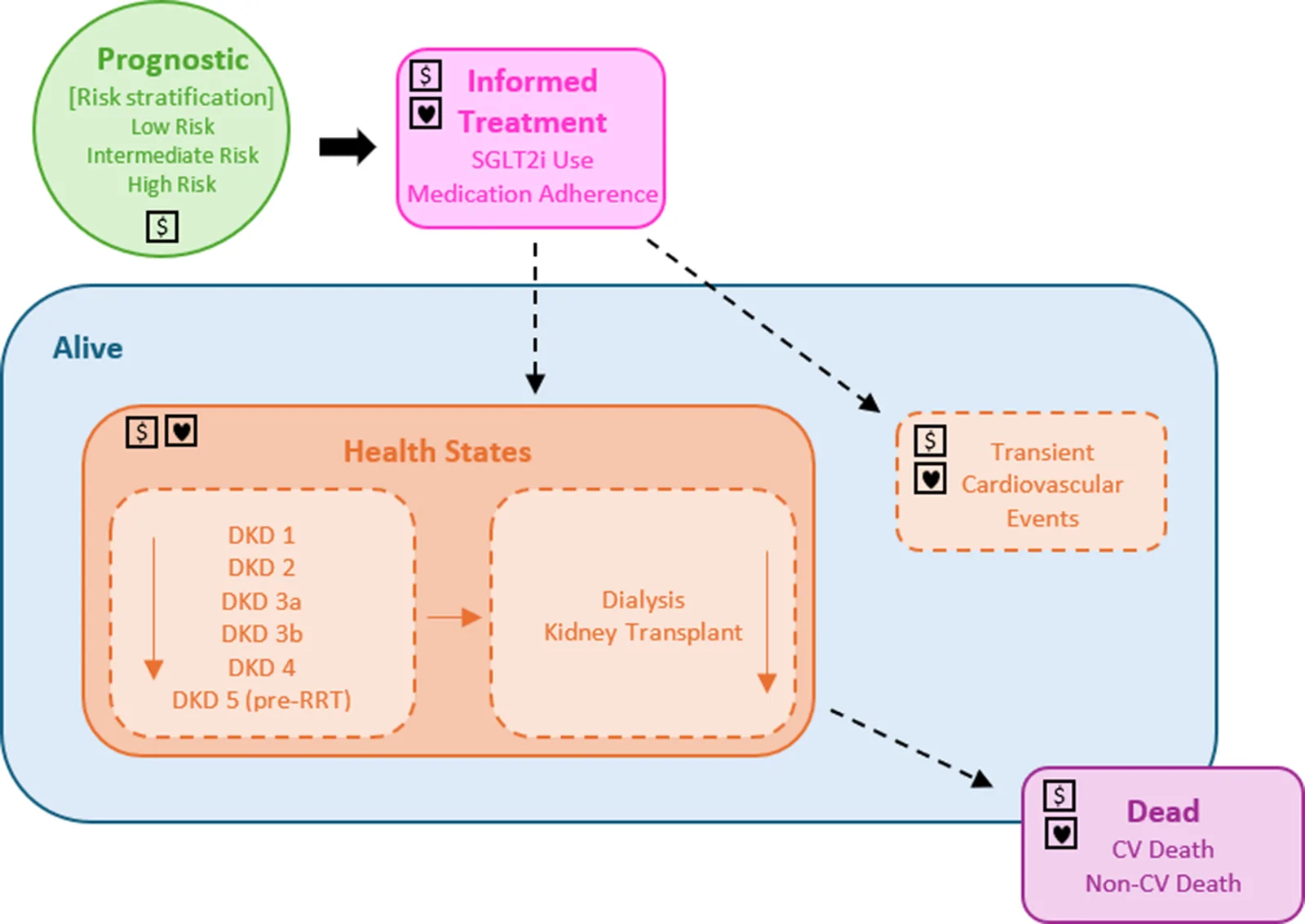
Model Framework. Sources: Developed by the authors for this work

Similar to KidneyIntelX, KDIGO-guided risk stratification using eGFR and uACR categorizes patients with stages 1-3b CKD into three risk groups: (1) “moderately increased risk” (2), “high risk”, and (3) “very high risk.” Standard testing through eGFR and UACR is hereafter referred to as “KDIGO”. A simple model framework is depicted in Fig. 1, which summarizes the patient pathway and direct impacts on outcomes from prognostic use.

The graphic showcases whether model components have an associated cost ($) or quality of life impact (❤) within each grouping. Patients begin the model by receiving a prognostic test (KidneyIntelX or KDIGO). This stratification informs utilization of SGLT2i treatment and also impacts patient adherence to this treatment. Medication utilization then has direct impacts on the frequency of cardiovascular events, as well as transition speed through health states, including DKD stage progression and advancement to dialysis or kidney transplant. DKD stage and utilization of dialysis or kidney transplant then dictate the patient’s mortality risk.

### Population

We modeled a hypothetical cohort of 100,000 patients with DKD in stages G1 or G2 with A2 or A3, or G3a-G3b with A1-A3 (based on the KDIGO classification shown as Fig. A1 in the Appendix (Additional File 1). KDIGO does not classify patients in stages G1 or G2 with A1 as having CKD and thus were excluded from the model population [20]. This cohort is expected to be representative of a standard DKD population, including any causes and contributors to DKD progression. At the start of the model, patients begin in stage G1 (excluding A1), G2 (excluding A2), G3a, or G3b based on prevalence estimates from a cross-sectional analysis of adults with T2D using National Health and Nutrition Examination Survey (NHANES) data [21]. Patients in the model receive Medicare coverage in the base case analysis and commercial health insurance coverage in a scenario analysis (Scenario 1).

Patients entering the model were assumed to have an average age of 65 years if receiving Medicare (base case analysis) and an average age of 60 years if receiving commercial health insurance (Scenario 1). Patients in the public payer perspective model received Medicare for the entirety of the lifetime model, and patients in the private payer perspective model received commercial health insurance for the entirety of the lifetime model.

Patients diagnosed with DKD Stages 1-3b were assumed to receive a prognostic screening test (KDIGO algorithm or KidneyIntelX) one time at the start of the model and were followed for the remainder of their lifetime. Patients experience progressive DKD in the model. Like other chronic kidney disease models, the model does not allow patients to experience improvements in eGFR between DKD stages, although we recognize that regression in CKD can occur in early stages [22–24]. 

### Model structure

**Fig. 2:**
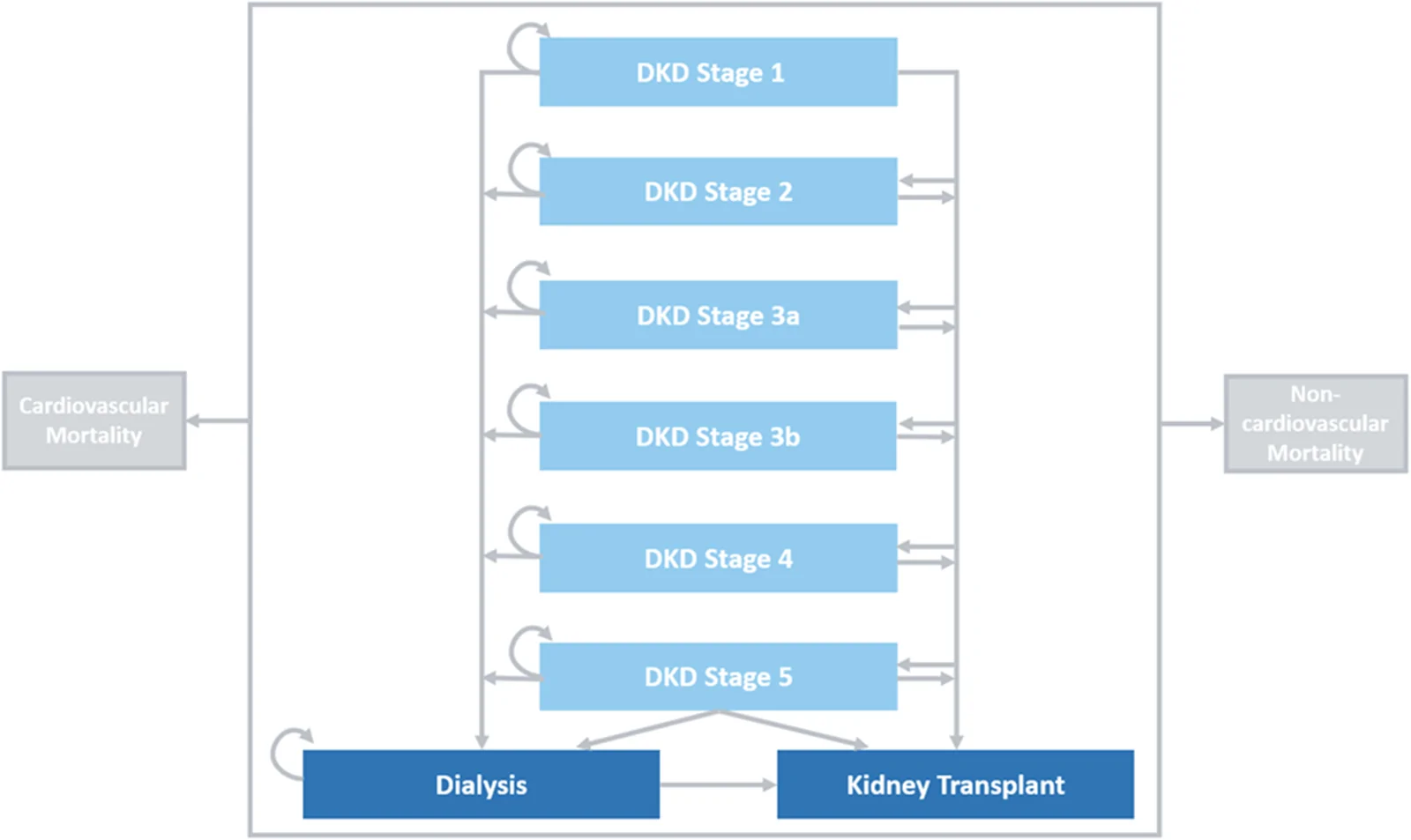
Markov model structure. Sources: Developed by the authors for this work

The CEA employs a Markov state transition structure (built in MS-Excel), which is a commonly used approach for economic analyses of CKD treatments and screening [25, 26]. The underlying structure of the model is a 10-state Markov model that accounts for testing outcomes (Fig. 2). States within the Markov model include Stage 1 (excluding A1), Stage 2 (excluding A2), Stage 3a, Stage 3b, Stage 4, Stage 5 (Pre-renal replacement therapy), Dialysis, Kidney Transplant, Cardiovascular Death, and Non-Cardiovascular Death. With each cycle, patients can transition forward in the model to any state or can remain in the existing state but cannot move backwards in DKD stage severity.

The model projects outcomes over a lifetime time horizon with a maximum patient age of 100 years old. Model cycle lengths are one year in length. All patients in the model receive the prognostic test (KidneyIntelX or KDIGO) one time upon cohort entry. Patients progress through stages 1–5 of DKD in the model, followed by end stage kidney disease (ESKD) requiring dialysis and/or kidney transplant. Costs and outcomes were discounted at 3% per year in the model, as recommended by the Public Health Service Panel on Cost Effectiveness in Medicine and supported by the Second Panel on Cost-Effectiveness in Health and Medicine [27]. 

### Transition probabilities

Patients in the model begin in one of the model stages representing DKD (stages 1, 2, 3a, or 3b), with the distribution informed by prevalence derived from National Health and Nutrition Examination Survey (NHANES) data [21]. Transition probabilities are informed by patient risk-group (low-, medium-, and high-risk) and corresponding eGFR. Distribution of KDIGO and KidneyIntelX patients across risk groups was based on the KidneyIntelX validation study [13]. All baseline structural parameters are reported in Table 1.

Median and average eGFR decline values were derived from biomarker and electronic health record (EHR) data of patients between the ages of 21–81 years with T2D and early stage DKD (Stages 1-3b) [13]. A validation dataset of 296 participants with actual non-imputed UACR values was analyzed to estimate eGFR decline among patient risk groups. Estimates of the median eGFR declines (ml/min/1.73m^2^) in the risk-groups of patients evaluated using KidneyIntelX were − 0.33, -2.00, -4.66 for low-, intermediate-, and high-risk groups, respectively. These eGFR declines were utilized to estimate the time within each state (considering single-state movement only) for both KidneyIntelX and KDIGO, as we assume that the treatment arm would have no influence on speed of progression before evaluation-driven treatment is experienced by patients. Median eGFR decline estimates were chosen as inputs (rather than average eGFR decline) because average decline values showed substantial variation due to outliers (i.e., very slow or very fast progressors). The time within each state was determined based on the annual rate of median eGFR decline and baseline eGFR values. The rate of transition was then calculated as 1/(time in state). Rates of transition were then converted to annual transition probabilities for each risk group and test.

**Table 1:** Baseline model parameters

Input	Base case Value	Source
Hypothetical screening population	100,000	NA
Distribution of patients with T2D across CKD stages		
CKD stage 1b (excluding A1), *n* (%)	28,000 (28%)	[21]
CKD stage 2 (excluding A2), *n* (%)	25,000 (25%)	[21]
CKD stage 3a, *n* (%)	31,000 (31%)	[21]
CKD stage 3b, *n* (%)	16,000 (16%)	[21]
Distribution of patients across risk-groups		
Low Risk, *n* (%)	45%	[13]
Intermediate Risk, *n* (%)	38%	[13]
High Risk, *n* (%)	17%	[13]
Percentage of dialysis starts that are dialysis crashes	29%	
Reduction in DKD progression due to SGLT2i’s	37%	[28]
Reduction in all-cause mortality due to SGLT2i’s	15%	[28]

Sources: Compiled by the authors based on values from the cited sources

In cases where data from the validation study were not available, transition data were supplemented by data from the U.S. Veterans Administration (VA), which includes a relatively large population of individuals with CKD or DKD. Specifically, VA data were used to estimate transition probabilities that did not progress linearly through the model (i.e., stages were jumped). The transition matrix shown in Table 2 below demonstrates which transition probabilities were informed by the validation study, and which were informed using VA data [28]. The probability of remaining in the index stage at the end of a cycle was calculated as 1 minus the aggregate probability of moving out of the index stage and varied based on age due to fluctuation in mortality rates over time.

**Table 2:** Transition matrix sources

Stage	Index Stage						
	S1	S2	S3a	S3b	S4	S5	DLS
S1	Calculated						
S2	Validation	Calculated					
S3a	VA data	Validation	Calculated				
S3b	VA data	VA data	Validation	Calculated			
S4	VA data	VA data	VA data	Validation	Calculated		
S5	VA data	VA data	VA data	VA data	Validation	Calculated	
DLS	VA data	VA data	VA data	VA data	Validation	Validation	Validation
KT	VA data	VA data	VA data	VA data	VA data	VA data	Validation

Sources: Developed by the authors for this work

The VA data transition probabilities required adjustment due to expected differences in transition timing between the VA population and the general DKD population. Data were also adjusted to allow for risk stratification between low-, intermediate-, and high-risk groups, utilizing the baseline VA data for the intermediate risk group. The original VA data are shown in the Appendix (Table A1) and resulting baseline transition probabilities (before treatment impacts were applied) are shown in Table A2.

Patients transitioning into the dialysis state were categorized as either crashers or non-crashers. A dialysis crash start is generally defined as a dialysis start occurring without previous nephrology care, often being initiated suddenly or in an inpatient setting [29]. The model assumes that 28% of patients crash onto dialysis, derived as an average of various source estimates ranging from 15% to 41.3%. This has two major impacts: (1) an increased first-year dialysis cost in comparison to non-crashers (see *Cost Estimates* for more detail), and (2) use of hemodialysis (HD) as opposed to peritoneal dialysis (PD) for the remainder of the patient’s time in the dialysis state. HD is expected to be used in 87% of non-crashing patients, with 13% of patients utilizing PD [30]. 

Background mortality by age was based on a 2019 life table from the National Vital Statistics System [31]. Mortality for each model state was then calculated for each age by applying a standardized mortality ratio to the mortality rates of the general US population. Evidence of mortality rates informing these calculations were collected from the 2018 United States Renal Data System (USRDS) annual report for the various DKD stages, while mortality for dialysis and kidney transplant patients were sourced from a retrospective cohort study of diabetic ESKD patients [32, 33]. The transition probabilities to death were divided between CV and non-CV death based on a cohort study of patients with incident ESKD, where 37.7% of all deaths in the population were due to CV-related causes [34]. Thus, we assumed 62.30% of deaths were due to non-cardiovascular causes. Death is an absorbing health state, and patients can transition to death (cardiovascular death or non-cardiovascular death) from any state. The annual transition probabilities to death at each age are reported in the Appendix (Table A3) alongside the standardized mortality ratios (SMRs) utilized for each model state.

### Treatment benefit

Evaluation from KidneyIntelX or KDIGO direct patient treatment pathways, captured in this model as SGLT2i use. Patients who received SGLT2i’s experienced benefits from treatment in the form of slowed DKD progression, reduced probability of death, and fewer CV events. The quantified impacts of SGLT2i use were informed by Neuen et al.’s meta-analysis of SGLT2i use on kidney outcomes [35]. For patients in Stages 1 to 3b who received KidneyIntelX, it was assumed that 7% of low-risk patients would be prescribed SGLT2i’s, 25% of intermediate-risk patients would be prescribed SGLT2i’s, and 50% of high-risk patients would be prescribed SGLT2i’s [36–38]. Patients in DKD stages 4 or 5 were assumed to experience the same SGLT2i utilization as standard care KDIGO-stratified patients. For patients using KDIGO risk stratification, SGLT2i utilization was assumed to vary by CKD stage rather than risk score. SGLT2i prescription rates were 18.2%, 18.4%, 15.5%, 6.6%, and 6.4% for patients in Stage 1, Stage 2, Stage 3a-b, Stage 4, and Stage 5, respectively [17]. In the base case analysis, intermediate-risk patients are attributed an adherence rate of 64% as reported for a diabetic population [39]. 

We then assume a distribution of 10% points around this mean value for low and high-risk KidneyIntelX patients, resulting in 54% adherence for low-risk and 74% adherence for high-risk patients in the model. We expect this variation to be lower for KDIGO patients, and thus varied adherence by 5% points to 59% and 69% for low- and high-risk KDIGO patients, respectively. In recognition of the absence of real-world adherence impacts, we also present a scenario analysis where the adherence to treatment for KidneyIntelX patients is equivalent to that of the KDIGO population (Scenario 3). For patients receiving the KidneyIntelX test and KDIGO test, all patients in intermediate or high-risk groups who are treated with SGLT2i’s experience a 37% decrease in progression (i.e., the transition probability from one stage to the next is reduced by 37%). Patients receiving SGLT2i’s in the low-risk category are assumed to experience no impact on disease progression from SGLT2i use. The resulting transition probabilities informing patient progression through the model are displayed in Table 3a-f.

**Table 3:** **a**-**f** Annual transition probabilities between health states across corresponding risk groups by prognostic screening type

Next	Index Stage						
	DKD Stage 1*	DKD Stage 2**	DKD Stage 3a	DKD Stage 3b	DKD Stage 4	DKD Stage 5	DLS
**a) Low risk KDIGO**							
DKD Stage 1*	97.45%						
DKD Stage 2**	2.14%	97.35%					
DKD Stage 3a	0.29%	2.14%	95.45%				
DKD Stage 3b	0.08%	0.41%	4.24%	95.53%			
DKD Stage 4	0.01%	0.07%	0.26%	4.24%	92.99%		
DKD Stage 5	0.00%	0.00%	0.01%	0.10%	4.24%	86.84%	
DLS	0.01%	0.01%	0.01%	0.09%	2.57%	12.20%	94.50%
KT	0.01%	0.01%	0.02%	0.05%	0.20%	0.96%	5.50%
**b) Intermediate risk KDIGO**							
DKD Stage 1*	90.67%	0	0	0	0	0	0
DKD Stage 2**	7.86%	90.28%	0	0	0	0	0
DKD Stage 3a	1.06%	7.86%	84.18%	0	0	0	0
DKD Stage 3b	0.30%	1.50%	14.74%	84.46%	0	0	0
DKD Stage 4	0.05%	0.27%	0.92%	14.74%	75.23%	0	0
DKD Stage 5	0.01%	0.01%	0.05%	0.34%	14.74%	62.58%	0
DLS	0.03%	0.03%	0.05%	0.30%	9.31%	34.68%	94.50%
KT	0.03%	0.04%	0.06%	0.16%	0.72%	2.74%	5.50%
**c) High risk KDIGO**							
DKD Stage 1*	80.03%	0	0	0	0	0	0
DKD Stage 2**	16.82%	79.21%	0	0	0	0	0
DKD Stage 3a	2.26%	16.82%	68.72%	0	0	0	0
DKD Stage 3b	0.65%	3.22%	29.15%	69.27%	0	0	0
DKD Stage 4	0.11%	0.59%	1.82%	29.15%	49.79%	0	0
DKD Stage 5	0.03%	0.03%	0.10%	0.67%	29.15%	42.57%	0
DLS	0.05%	0.06%	0.10%	0.60%	19.60%	53.23%	94.50%
KT	0.08%	0.13%	0.19%	0.49%	2.32%	6.67%	5.50%
**d) Low risk KidneyIntelX**							
DKD Stage 1*	97.45%						
DKD Stage 2**	2.14%	97.35%					
DKD Stage 3a	0.29%	2.14%	95.45%				
DKD Stage 3b	0.08%	0.41%	4.24%	95.53%			
DKD Stage 4	0.01%	0.07%	0.26%	4.24%	92.99%		
DKD Stage 5	0.00%	0.00%	0.01%	0.10%	4.24%	86.84%	
DLS	0.01%	0.01%	0.01%	0.09%	2.57%	12.20%	94.50%
KT	0.01%	0.01%	0.02%	0.05%	0.20%	0.96%	5.50%
**e) Intermediate risk KidneyIntelX**							
DKD Stage 1*	90.67%	0	0	0	0	0	0
DKD Stage 2**	7.86%	90.28%	0	0	0	0	0
DKD Stage 3a	1.06%	7.86%	84.18%	0	0	0	0
DKD Stage 3b	0.30%	1.50%	14.74%	84.46%	0	0	0
DKD Stage 4	0.05%	0.27%	0.92%	14.74%	75.23%	0	0
DKD Stage 5	0.01%	0.01%	0.05%	0.34%	14.74%	62.58%	0
DLS	0.03%	0.03%	0.05%	0.30%	9.31%	34.68%	94.50%
KT	0.03%	0.04%	0.06%	0.16%	0.72%	2.74%	5.50%
**f) High risk KidneyIntelX**							
DKD Stage 1*	80.03%	0	0	0	0	0	0
DKD Stage 2**	16.82%	79.21%	0	0	0	0	0
DKD Stage 3a	2.26%	16.82%	68.72%	0	0	0	0
DKD Stage 3b	0.65%	3.22%	29.15%	69.27%	0	0	0
DKD Stage 4	0.11%	0.59%	1.82%	29.15%	49.79%	0	0
DKD Stage 5	0.03%	0.03%	0.10%	0.67%	29.15%	42.57%	0
DLS	0.05%	0.06%	0.10%	0.60%	19.60%	53.23%	94.50%
KT	0.05%	0.08%	0.12%	0.31%	1.46%	4.20%	5.50%

*G1b or G1c

**G2b or G2c

There is also evidence that patients receiving SGLT2i’s experience a reduction in CV events compared to those not receiving SGLT2i’s. To incorporate this benefit, we utilized the rates of CV events for each eGFR level among DKD placebo patients from USRDS (stage 1 = 4.18%, stage 2 = 4.18%, stage 3a = 5.11%, stage 3b = 5.84%, stage 4 = 12.21%, stage 5 = 12.21%) as baseline values, assuming that these event rates occur in our base population before SGLT2i use [40]. We then calculated a reduction in CV events from the baseline using the estimated SGLT2i impact (a 36% reduction compared to placebo) for patients administered SGLT2i’s in each arm [35]. Resulting CV event rates are reported in Table 4. Patients receiving SGLT2i’s also experience a 15% reduction in all-cause mortality [35]. CV event probability and mortality rates are unaffected by SGLT2i use for patients in the low-risk category.

**Table 4:** CV events

Disease State	CV Event Rate		
	Low Risk	Intermediate Risk	High Risk
DKD Stage 1			
KidneyIntelX	4.18%	3.94%	3.62%
KDIGO	4.18%	4.00%	3.99%
DKD Stage 2			
KidneyIntelX	4.18%	3.94%	3.62%
KDIGO	4.18%	4.00%	3.99%
DKD Stage 3a			
KidneyIntelX	5.11%	4.82%	4.43%
KDIGO	5.11%	4.93%	4.91%
DKD Stage 3b			
KidneyIntelX	5.84%	5.50%	5.06%
KDIGO	5.84%	5.63%	5.62%
DKD Stage 4			
KidneyIntelX	12.21%	12.02%	12.00%
KDIGO	12.21%	12.02%	12.00%
DKD Stage 5			
KidneyIntelX	12.21%	12.03%	12.00%
KDIGO	12.21%	12.03%	12.00%

Sources: Compiled by the authors for this work

Recent evidence suggests that a novel mineralocorticoid receptor antagonist (MRA), finerenone, may be an effective treatment for renal patients [41]. Treatment benefit from MRAs in addition to SGLT2i use was considered as a scenario analysis (Scenario 2). Recent evidence shows notable reductions in CV event rates (50%), all-cause mortality probability (24%), and overall DKD progression (51%) for patients utilizing SGLT2i and MRAs compared to patients taking placebo [35]. The addition of MRAs was also found to improve outcomes compared to SGLT2i use alone. These benefits were applied in Scenario 2 as reductions from the baseline values reported in the base case scenario. CV event rates and DKD transition probabilities reflecting the impact of combined SGLT2i and MRA use for each prognostic arm are shown in Appendix Table A4.

### Cost estimates

Cost inputs used in this model include the costs of testing, medication, and office visits, as well as annual disease state costs for each DKD stage, dialysis, and kidney transplant. All economic inputs were derived from targeted literature searches. Input values and aligning sources are reported in 2024 USD in Table 5. The cost of KDIGO testing is assumed to include the costs of an eGFR and UACR test, and a $100 administration cost is assumed. The reported cost of KidneyIntelX was obtained from the previously published budget-impact analysis and reflects the pricing from the Medicare fee schedule and also includes the one-time administration cost [10]. As we assume that KidneyIntelX will be used with eGFR and UACR (but not necessarily their risk categories), the cost of KDIGO is added to the reported KidneyIntelX cost when used in the model. In the base case analysis, the annual cost of each stage of DKD were averaged across three different studies reporting Medicare costs, which did not include dialysis or kidney transplant costs as these costs were accounted for in separate model states [12, 42, 43]. In Scenario 1, commercial costs for CKD stages were averaged between two sources [12, 44]. All other costs were assumed to be the same between insurance types. The annual costs of HD and PD were obtained from the USRDS 2021 annual report and the reported cost of dialysis represents a weighted average of the two methods [45]. Crashing onto dialysis was estimated to add $72,412 to first-year dialysis costs [46]. Costs for a kidney transplant, post-transplant care, CV death-related end of life care, and non-CV death-related end of life care were all obtained from literature [7, 47, 48]. 

**Table 5:** Economic parameters

Input	Base case Value	Source
Test Inputs		
Cost per KidneyIntelX test	$980	[10]
Administrative cost of KidneyIntelX	$100	[10]
Cost of KDIGO test	$30	Assumption
Administrative cost of KDIGO test	$100	Assumption
Annual cost per patient at each CKD stage (Medicare)*		
CKD Stage 1	$17568.15	[12, 42, 43]
CKD Stage 2	$20804.77	[12, 42, 43]
CKD Stage 3a	$22020.02	[12, 42, 43]
CKD Stage 3b	$28813.51	[12, 42, 43]
CKD Stage 4	$38872.58	[12, 42, 43]
CKD Stage 5	$50865.33	[12, 42, 43]
Annual cost per patient at each CKD stage (Commercial)**		
CKD Stage 1	$23514.48	[12, 44]
CKD Stage 2	$24890.57	[12, 44]
CKD Stage 3a	$31594.65	[12, 44]
CKD Stage 3b	$51257.43	[12, 44]
CKD Stage 4	$66037.58	[12, 44]
CKD Stage 5	$139095.73	[12, 44]
Event Costs		
Annual cost of dialysis	$112617.04	[45]
Cost of a dialysis crash	$185029.72	[46]
Cost of kidney transplant	$280830.73	[47]
Annual cost of post-transplant care	$42874.92	[48]
Cost of a CV event	$30846.51	[7]
Cost of cardiovascular death	$20642.23	[7]
Cost of non-cardiovascular death	$13638.47	[7]

*Used in base-case

**Used in scenario 1

Sources: Developed by the authors based on values from the cited sources

CV event costs reflect the costs of heart failure, stroke, and myocardial infarction in a DKD population [7]. Weighting the individual event costs by the event rate over a 4-month period results in an average CV event cost of $30,846.51. Costs of preventative services including monitoring and managing CKD progression were incorporated into the model for the DKD stage disease states under the assumption that the annual number of office visits would vary depending on DKD stage. The use of KidneyIntelX would further drive this count, increasing the portion of patients referred for high-risk patients (but not causing any impact on disease progression or quality of life). The resulting probabilities of office visits can be found in Appendix A (Table A5). Visit types included nephrologist evaluations, dietitian meetings, CKD education services, and behavioral assessments. Costs for each type of visit were based on CPT coding and were averages across facility and non-facility fees [22]. Medication costs were also considered in the model, although SGLT2i’s alone had any differential impact on patient outcomes. The cost for SLGT2 inhibitors was obtained from GoodRx and reflected the monthly cost of 10 mg doses of Dapagliflozin [49]. Costs and prescription rates (with adherence impacts) for all other medications can be found in the Appendix (Table A6) and were sourced from Medi-Span’s Price Rx resource, with values inflated to 2024 USD according to the available medication-based consumer price index reports [50]. 

### Utilities

Utility data for DKD stages were derived from a study conducted on individuals who were both members of a large research cohort of veterans with diabetes and who responded to the Large Veterans Health Survey (LVHS) in 1999 [51]. Pre-existing SF-12 responses were transformed to EQ-5D values. We used the mean utility values from a subset of the dataset without mental illness or substance-abuse disorders and were defined as prevalent DKD cases, meaning they had diabetes mellites for at least 3 years. A potential limitation of this source was that most participants were men (> 97%). Utility values for kidney transplant in year 1 and kidney transplant maintenance (years 2+) were incorporated into the model based on a cost-effectiveness model by Howard et al. (2010) [52, 53]. It was assumed that patients who “crashed” onto dialysis experienced a lower health utility value in year 1 (utility = 0.615) compared to those who begin dialysis without experiencing a dialysis crash. All utility values are reported alongside data sources in Table 6.

**Table 6:** Utility values

Input Parameter	Value	Source
DKD Stage 1	0.715	[51]
DKD Stage 2	0.714	(51)
DKD Stage 3a	0.692	[51]
DKD Stage 3b	0.666	[51]
DKD Stage 4	0.649	[51]
DKD Stage 5	0.633	[51]
Year 1 Dialysis (Crash)	0.615	Assumption
Year 1 Dialysis (Non-Crash)	0.623	Assumption
ESRD/Dialysis	0.623	[51]
Kidney Transplant Year 1	0.73	[52, 53]
Kidney Transplant Follow-Up Years	0.70	[52, 53]
CV Death	0.00	-
Non-CV Death	0.00	-

Sources: Developed by the authors based on values from the cited sources

### Outcomes

The primary outcomes of the cost-effectiveness analysis are life years, quality adjusted life years (QALYs), lifetime costs, incremental cost effectiveness ratios (ICERs), and net monetary benefit (NMB). Secondary outcomes included months spent in each stage of DKD, CV events avoided, and dialysis crashes and ESKD events, including total number of dialysis starts and total number of kidney transplants. Outcomes were summarized for the base case analysis as well as the scenario analysis.

The ICER represented in terms of dollars/QALYs, is a comparative measure typically used in the economic analysis of healthcare data and represents the comparative value of an intervention. When an ICER falls below the willingness-to-pay (WTP) threshold, then it is typically considered a cost-effective intervention compared with its modeled comparator. ICERs are calculated as follows:

$$\:\mathrm{I}\mathrm{C}\mathrm{E}\mathrm{R}=\:\frac{{\mathrm{C}\mathrm{o}\mathrm{s}\mathrm{t}}_{\mathrm{I}\mathrm{n}\mathrm{t}\mathrm{e}\mathrm{r}\mathrm{v}\mathrm{e}\mathrm{n}\mathrm{t}\mathrm{i}\mathrm{o}\mathrm{n}}-{\mathrm{C}\mathrm{o}\mathrm{s}\mathrm{t}}_{\mathrm{C}\mathrm{o}\mathrm{m}\mathrm{p}\mathrm{a}\mathrm{r}\mathrm{a}\mathrm{t}\mathrm{o}\mathrm{r}}}{{\mathrm{Q}\mathrm{A}\mathrm{L}\mathrm{Y}}_{\mathrm{I}\mathrm{n}\mathrm{t}\mathrm{e}\mathrm{r}\mathrm{v}\mathrm{e}\mathrm{n}\mathrm{t}\mathrm{i}\mathrm{o}\mathrm{n}}-{\mathrm{Q}\mathrm{A}\mathrm{L}\mathrm{Y}}_{\mathrm{c}\mathrm{o}\mathrm{m}\mathrm{p}\mathrm{a}\mathrm{r}\mathrm{a}\mathrm{t}\mathrm{o}\mathrm{r}}}$$

A WTP of $100,000 is typical in US cost-effectiveness analyses. This threshold is then used to calculate the NMB of the intervention versus the comparator using the following equation:

$$\eqalign{ {\rm{NMB}} = & {\rm{Incremental}}\,{\rm{QALYs}} \times {\rm{WTP}} \cr& - {\rm{Incremental}}\,{\rm{Cost}} \cr}$$

A probabilistic sensitivity analysis (PSA) was conducted to support the KidneyIntelX CEA. The probabilistic sensitivity analysis functions as a Monte Carlo simulation where each variable is varied according to its given distribution, mean value, and standard error. When standard error was not available, 10% of the deterministic value was assumed. Model results are then recorded for a set number of iterations. The results of these recorded iterations allow us to determine the likelihood that an intervention is cost-effective relative to the WTP threshold.

## Results

### Base case

Results of the base case analysis report clinical endpoints and economic savings from a Medicare perspective assuming an average patient age of 65. Clinical and economic outcomes for the base case analysis are displayed in Table 7.

**Table 7:** Base case results

	KidneyIntelX	KDIGO	Delta (Δ)
Clinical Outcomes			
Dialysis Crashes	8396	8482	-86
Total Dialysis Starts	29,305	29,604	-299
Kidney Transplants	7671	7784	-113
Total Patients Initiating ESKD	36,976	37,388	-412
Months Spent in DKD 1	2.181	2.164	0.017
Months Spent in DKD 2	2.611	2.588	0.023
Months Spent in DKD 3a	2.691	2.663	0.028
Months Spent in DKD 3b	2.486	2.456	0.030
Months Spent in DKD 4	0.919	0.932	-0.013
Months Spent in DKD 5	0.294	0.299	-0.005
LYs	8.701	8.663	0.038
QALYs	5.960	5.932	0.028
Economic Outcomes			
Costs	$514107.83	$514621.62	-$513.79
DKD Stage & ESRD Costs	$499404.58	$501214.50	-$1809.93
CV Event Savings	$480.11	$268.43	$211.68
Office Visit & Medication Costs	$14103.36	$13545.54	$557.82
Test Costs	$1080.00	$130.00	$950.00
NMB	$81929.06	$78618.42	$3310.64
ICER ($/QALY)	**-$18370.26**		

Note: Base case reflects Medicare beneficiaries ages 65 + with SGLT2i use directed by KidneyIntelX or KDIGO testing

Sources: Compiled by the authors for this work

**Fig. 3:**
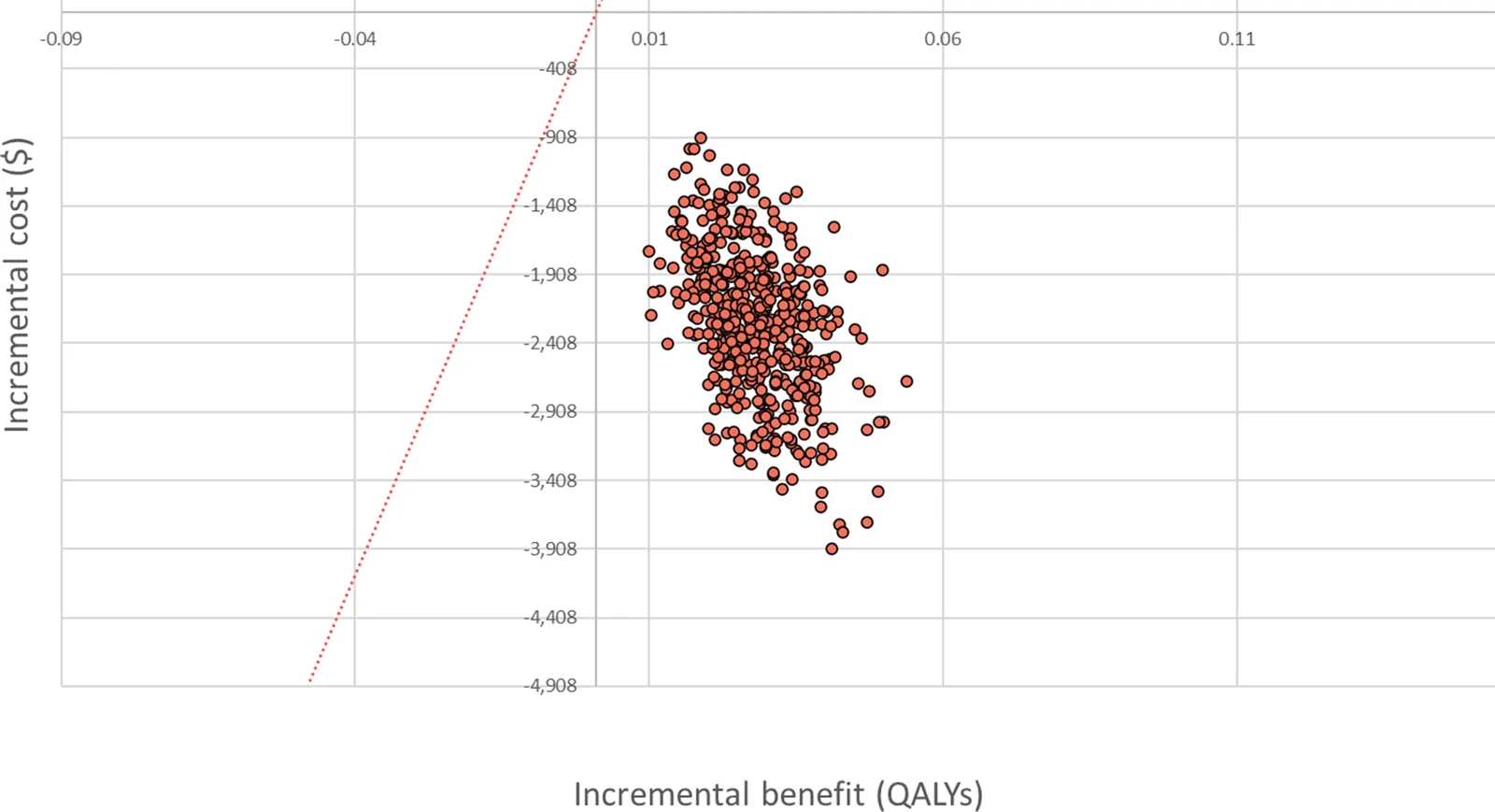
Base Case PSA Scatterplot. Sources: Developed by the authors based on model results

The clinical results show that the use of KidneyIntelX would lead to a reduction in total number of patients initiating ESKD treatment, including dialysis starts, dialysis crashes, and kidney transplants. KidneyIntelX patients would also spend less time in the more severe DKD stages (4 and 5) and more time in earlier stages (1 through 3b). These patients therefore experience lower DKD and ESRD treatment costs than KDIGO patients. KidneyIntelX is also expected to increase patient lifespan, thereby increasing patient QALYs in comparison to KDIGO. With decreased lifelong costs resulting in savings of $514 per patient, KidneyIntelX is expected to dominate KDIGO with a negative ICER (-$18,370) due to lower costs and improved quality of life. The scatterplot of 500 PSA simulations (Fig. 3) shows that with reasonable variation in model parameters, KidneyIntelX remains a dominant strategy in all simulations, with ICERs remaining below the $100,000 willingness to pay ICER threshold.

### Scenario analysis 1: commercial perspective

The clinical and economic results for Scenario 1 are reported in Table 8. These outcomes reflect a commercial payer perspective with an average patient age of 54.

**Table 8:** Scenario 1 results

	KidneyIntelX	KDIGO	Delta (Δ)
Clinical Outcomes			
Dialysis Crashes	11,435	11,496	-62
Total Dialysis Starts	39,912	40,127	-215
Kidney Transplant	13,963	14,110	-146
Total Patients Initiating ESKD	53,875	54,237	-362
Months Spent in DKD 1	2.832	2.813	0.020
Months Spent in DKD 2	3.531	3.502	0.029
Months Spent in DKD 3a	3.536	3.501	0.035
Months Spent in DKD 3b	3.467	3.426	0.040
Months Spent in DKD 4	1.421	1.435	-0.015
Months Spent in DKD 5	0.458	0.464	-0.006
LYs	12.231	12.195	0.036
QALYs	8.349	8.322	0.027
Economic Outcomes			
Costs	$829762.83	$831246.46	-$1483.63
DKD Stage & ESRD Costs	$811188.86	$813674.81	-$2485.95
CV Event Savings	$570.68	$325.65	$245.03
Office Visit & Medication Costs	$18064.65	$17767.30	$297.35
Test Costs	$1080.00	$130.00	$950.00
NMB	$5168.49	$943.37	$4225.12
ICER ($/QALY)			**-$54117.51**

Note: S1 reflects all base-case assumptions, but with a cohort of commercial payer beneficiaries ages 54+

Sources: Compiled by the authors based on model results

**Fig. 4:**
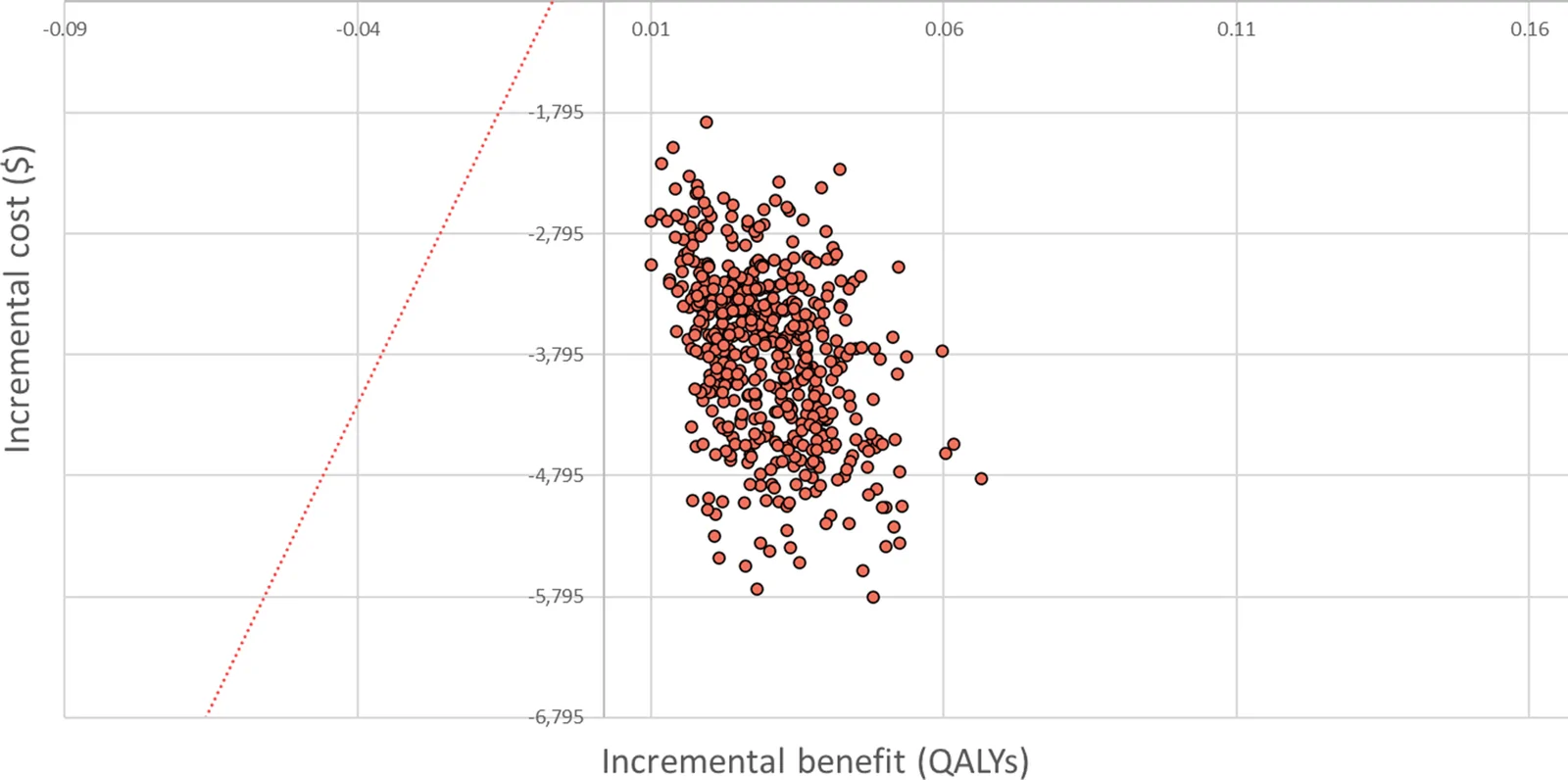
Scenario 1 PSA Scatterplot. Sources: Developed by the authors based on model results

Results show reductions in ESKD treatment initiation, an increase in total life years, increase in total QALYs, and decreased costs over a lifetime using KidneyIntelX. Lower lifetime costs of about $1,484 and QALY gains of 0.027 per patient result in a dominant ICER of -$54,118. PSA output (Fig. 4) shows that these results remain robust with input variation, staying cost-effective at a $100,000 WTP threshold.

### Scenario analysis 2: SGLT2 inhibitors + MRAs

**Fig. 5:**
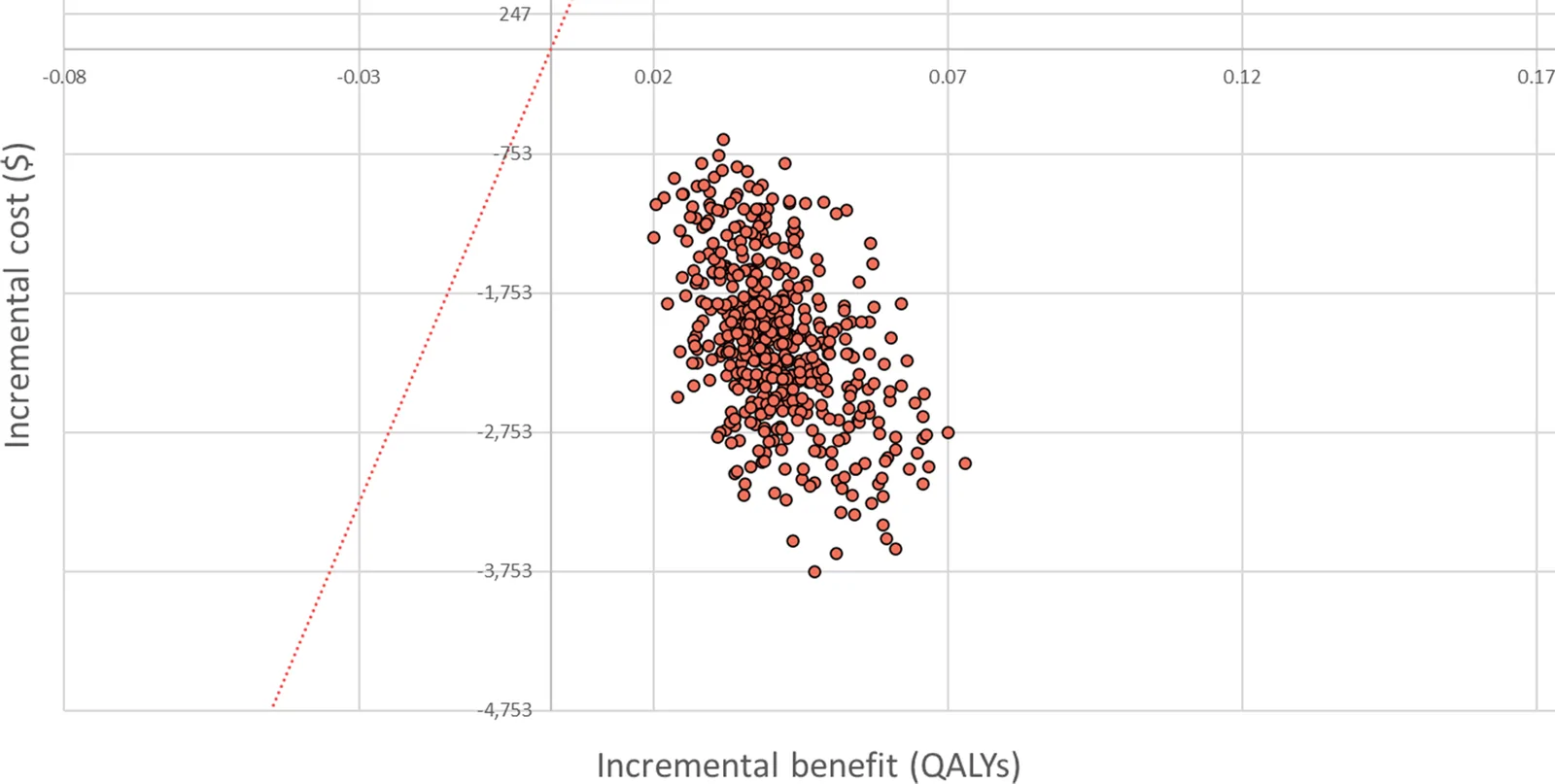
Scenario 2 PSA Scatterplot. Sources: Developed by the authors based on model results

Scenario 2 implements the use of MRAs alongside SLGT2 inhibitor use for patients directed to treatment, assuming the Medicare perspective and an average patient age of 65. Results (Table 9) show that utilizing MRAs in addition to SGLT2i’s increases the number of QALYs gained (0.039) and the cost savings ($530) for patients using KidneyIntelX compared to KDIGO alone. These outcomes lead to KidneyIntelX + KDIGO being a dominant strategy compared to KDIGO alone when the impact of MRA use is applied. The PSA (Fig. 5) shows that all simulation runs result in an ICER that fall into the southeast quadrant of the plane, indicating that KidneyIntelX both saves money and adds QALYs compared to KDIGO.

**Table 9:** Scenario 2 Results

	KidneyIntelX	KDIGO	Delta (Δ)
Clinical Outcomes			
Dialysis Crashes	8350	8475	-125
Total Dialysis Starts	29,144	29,581	-437
Kidney Transplant	7616	7777	-160
Total Patients Initiating ESKD	36,761	37,358	-597
Months Spent in DKD 1	2.189	2.165	0.024
Months Spent in DKD 2	2.622	2.589	0.032
Months Spent in DKD 3a	2.702	2.664	0.037
Months Spent in DKD 3b	2.498	2.458	0.040
Months Spent in DKD 4	0.915	0.931	-0.016
Months Spent in DKD 5	0.292	0.298	-0.006
LYs	8.718	8.666	0.052
QALYs	5.973	5.934	0.039
Economic Outcomes			
Costs	$514278.07	$514807.68	-$529.61
DKD Stage & ESRD Costs	$498627.30	$501112.28	-$2484.97
CV Event Savings	$606.67	$287.04	$319.63
Office Visit & Medication Costs	$15177.43	$13852.44	$1324.99
Test Costs	$1080.00	$130.00	$950.00
NMB	$82996.32	$78603.12	$4393.19
ICER ($/QALY)			**-$13707.63**

Note: S2 reflects all base-case assumptions but adds the impact of KidneyIntelX- or KDIGO-directed MRA use on CV events

Sources: Developed by the authors based on model results

### Scenario analysis 3: equivalent adherence

**Fig. 6:**
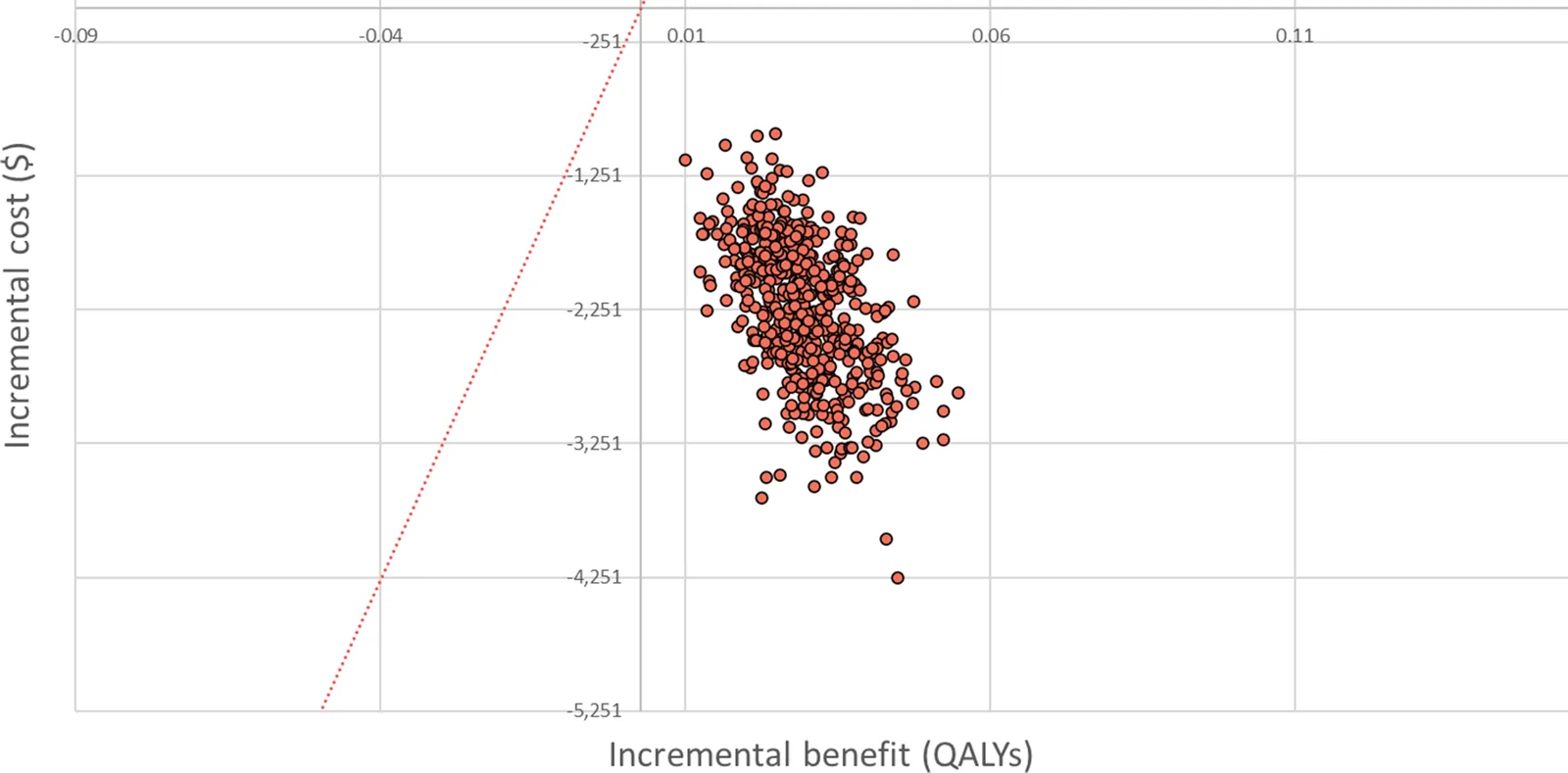
Scenario 3 PSA Scatterplot. Sources: Developed by the authors based on model results

Scenario 3 utilizes all base-case assumptions (including an average age of 65 and a Medicare population), while removing the potential impact from KidneyIntelX on adherence rates. We therefore assume that KidneyIntelX adherence rates are equivalent to the KDIGO-guided treatment adherence rates of 59%, 64%, and 69% for low-, intermediate-, and high-risk patients. The results displayed in Table 10 indicate that we could still expect a dominant outcome without adherence impacts, where KidneyIntelX patients experienced increased LYs and QALYs and reduced overall costs compared to KDIGO patients. As shown in the PSA scatterplot in Fig. 6, these results remain robust to reasonable variations in input parameters.

**Table 10:** Scenario 3 results

	KidneyIntelX	KDIGO	Delta (Δ)
Clinical Outcomes			
Dialysis Crashes	8403	8481	-78
Total Dialysis Starts	29,331	29,603	-272
Kidney Transplant	7681	7784	-103
Total Patients Initiating ESKD	37,012	37,387	-375
Months Spent in DKD 1	2.180	2.164	0.016
Months Spent in DKD 2	2.609	2.588	0.021
Months Spent in DKD 3a	2.689	2.663	0.027
Months Spent in DKD 3b	2.485	2.456	0.028
Months Spent in DKD 4	0.919	0.932	-0.013
Months Spent in DKD 5	0.294	0.299	-0.005
LYs	8.698	8.663	0.035
QALYs	5.958	5.932	0.026
Economic Outcomes			
Costs	$514250.60	$514619.94	-$369.34
DKD Stage & ESRD Costs	$499559.00	$501212.84	-$1653.83
CV Event Savings	$466.32	$268.43	$197.89
Office Visit & Medication Costs	$14077.92	$13545.53	$532.38
Test Costs	$1080.00	$130.00	$950.00
NMB	$81589.79	$78618.66	$2971.13
ICER ($/QALY)			**-$14195.57**

Note: S3 reflects all base-case assumptions but removes the treatment adherence differential between KidneyIntelX and KDIGO patients

Sources: Developed by the authors based on model results

## Discussion

The goal of this analysis was to determine the cost-effectiveness of utilizing KidneyIntelX compared to KDIGO by estimating the incremental economic cost and incremental QALY benefit of each diagnostic method. The study demonstrated that the use of KidneyIntelX is associated with substantial clinical benefits from slowed disease progression which manifested in the model as more time spent in low severity DKD stages and less time spent in high severity DKD states, leading to fewer dialysis starts and kidney transplants in the study population in addition to fewer CV events. As such, patients in both the main and scenario analysis are expected to experience QALY gains compared to the KDIGO-diagnosed population.

The results of the analysis show increased costs and small QALY gains for KidneyIntelX compared to KDIGO. The magnitude of these outcomes is favorable when compared to other prognostic approaches in the CKD disease space, which report nearly negligible, positive QALY gains resulting from diagnostic testing [54, 55]. For example, a screening test for CKD based on eGFR estimated QALY gains of 0.0044 QALYs per patient in the Canadian population, a considerably smaller improvement in quality of life when compared to the 0.028 gain expected for the KidneyIntelX risk-scored population [55]. 

Slowed disease progression triggered by earlier identification and risk-driven treatment using KidneyIntelX is expected to have a significant impact on the DKD population. As various studies have demonstrated, the cost of DKD management increases with disease progression [56–58]. Recently, the US government has emphasized the need for novel approaches to slowing DKD progression or prevention [59]. Various treatments currently exist which address disease management, including this study’s main vehicle for treatment benefit, SGLT2i’s. Although initially developed to manage hyperglycemia, SGLT2i’s have proven to be successful in reducing eGFR and slowing disease progression [59]. However, a major barrier to successful improvements in CKD is underdiagnosis, which leads to a large portion of untreated or undertreated patients [60]. This treatment gap is what KidneyIntelX aims to fill, by helping to facilitate the identification of early-stage DKD and to promote the use of guideline directed care and treatment plans that reflect an individual patient’s potential for a progressive decline in their kidney function. It is anticipated that this approach will also serve to address the risk-treatment paradox seen in clinical practice [61]. It is likely that this type of prognostic tool will lead to reductions in disease progression, increases in life span, and potential cost savings throughout the DKD patient lifespan.

## Conclusions

This analysis assessed the cost-effectiveness of KidneyIntelX as a prognostic test for the assessment of chronic / diabetic kidney disease progression in comparison to prognosis relying on KDIGO. Our results demonstrate that the average Medicare patient population may experience fewer CV events, spend more time in less severe DKD disease stages, and experience fewer dialysis (emergency) starts and kidney transplants by using KidneyIntelX compared to KDIGO. These clinical impacts could lead to an increased life span and quality-adjusted life span for patients using KidneyIntelX. With incremental costs of approximately $514 per patient from the Medicare perspective and incremental QALYs of 0.028, KidneyIntelX would likely be a cheaper prognostic approach compared to KDIGO and could lead to increased quality-adjusted life years. The resulting ICER is negative, meaning that KidneyIntelX may be a dominant strategy compared to KDIGO. Results are similar from the commercial perspective considered in the scenario analysis, cost savings of about $1,484 patient and QALY gains of 0.027, resulting in a dominant, negative ICER. KidneyIntelX is also dominant from the Medicare perspective compared to KDIGO when the use of MRAs in addition to SGLT2i’s is modeled, with cost savings of $530 per patient and QALY gains of 0.039 over a lifetime. Finally, when no impact from KidneyIntelX on treatment adherence is modeled, KidneyIntelX still shows a dominant ICER with $369 in cost savings and 0.026 added QALYs.

This study has several limitations to discuss. First, many of the input values were sourced from literature and thus may not reflect the exact population existing in the KidneyIntelX clinical study. For instance, reduction in disease progression due to SGLT2i use was estimated from several articles reporting benefits within this range, which were not DKD-specific [14, 35, 62–64]. Similarly, disease progression data were supplemented with evidence from a CKD/DKD veteran population which may not represent the general DKD population. However, a complete stochastic sensitivity analysis was conducted which demonstrated that variation within the model inputs’ reasonable parameters does not disrupt the clinical or economic benefit of KidneyIntelX use. As a cohort-based analysis, the model assumed an average patient age and CKD stage at diagnosis. It is emphasized that these factors will vary in a real-world setting. Further, a causal relationship between the use of KidneyIntelX and reduced disease progression cannot be confirmed, but current evidence supports that earlier identification and treatment would delay disease progression and improve patient outlook. These impacts are also dependent on clinician adherence to KidneyIntelX-guided treatment, which will vary in a real-world setting. Finally, this model did not quantify the impact of indirect costs such as absenteeism, presenteeism, or caregiver burden. With these drivers omitted, we expect this analysis to be a conservative estimation of the overall societal impact that KidneyIntelX may be expected to have on the DKD population.

While this study provides an analysis-based estimate of the potential cost-effectiveness of KidneyIntelX, it is a simulation-based model which does not capture real-world healthcare utilization data. Future studies would be appropriate to capture patient outcomes when used in a real-world setting. Clinical outcomes to consider include patient QoL (captured through EQ-5D or similar), medication utilization, healthcare visits, and total healthcare costs. Patient-level outcomes from this type of study would add to the body of evidence for KidneyIntelX, providing validation or contradicting evidence to its efficacy and cost-effectiveness.

## Electronic Supplementary Material

Below is the link to the electronic supplementary material.

Supplementary Material 1

## Data Availability

No datasets were generated or analysed during the current study.

## Abbreviations

BIM: Budget impact model
CDC: Centers for Disease Control and Prevention
CEA: Cost-effectiveness analysis
CKD: Chronic kidney disease
CV: Cardiovascular
DKD: Diabetic kidney disease
eGFR: Estimated glomerular filtration rate
EHR: Electronic health record
ESKD: End stage kidney disease
KDIGO: Standard testing through eGFR and UACR
HD: Hemodialysis
HRQoL: Health-related quality of life
ICER: Incremental cost effectiveness ratio
KDIGO: Kidney Disease Improving Global Outcomes
LVHS: Large Veterans Health Survey
MRA: Mineralocorticoid receptor antagonist
NHANES: National Health and Nutrition Examination Survey
NMB: Net monetary benefit
PD: Peritoneal dialysis
PDKF: Progressive decline in kidney function
PSA: Probabilistic sensitivity analysis
QALY: Quality adjusted life year
SGLT2i: Sodium-glucose co-transporter-2 inhibitors
SMR: Standardized mortality ratio
SOC: Standard of care
T2D: Type 2 diabetes
UACR: Urine albumin-creatinine ratio
US: United States
VA: U.S. Veterans Administration
WTP: Willingness to pay
