# Supplementary material for: Cost-effectiveness analysis of a prognostic risk assessment for early-stage 1-3b diabetic kidney disease patients in the United States
Source: Cost Eff Resour Alloc. 2026 Jun 24;24:112. doi: 10.1186/s12962-026-00775-4 (PMC13548659; doi:10.1186/s12962-026-00775-4)
Supplement: Supplementary file 1 [file 12962_2026_775_MOESM1_ESM.docx]

**Appendix**

**Table A1.** VA Transition Probabilities

| Next | Index Stage | | | | | | |
| --- | --- | --- | --- | --- | --- | --- | --- |
|  | **DKD Stage 1*** | **DKD Stage 2**** | **DKD Stage 3a** | **DKD Stage 3b** | **DKD Stage 4** | **DKD Stage 5** | **DLS** |
| DKD Stage 1* | 83.57% |  |  |  |  |  |  |
| DKD Stage 2** | 14.36% | 87.38% |  |  |  |  |  |
| DKD Stage 3a | 0.88% | 9.95% | 85.48% |  |  |  |  |
| DKD Stage 3b | 0.25% | 1.26% | 10.43% | 83.08% |  |  |  |
| DKD Stage 4 | 0.04% | 0.23% | 0.77% | 10.81% | 76.68% |  |  |
| DKD Stage 5 | 0.01% | 0.01% | 0.04% | 0.28% | 7.87% | 55.96% |  |
| DLS | 0.02% | 0.02% | 0.04% | 0.25% | 6.01% | 31.26% | 79.11% |
| *G1b or G1c  **G2b or G2c |  |  |  |  |  |  |  |

**Table A2.** Annual Transition Probabilities between Health States Across Corresponding Risk Groups (Baseline values before SGLT2i impact)

|  | Low Risk | Intermediate Risk | High Risk |
| --- | --- | --- | --- |
| G1* to G2** | 2.14% | 12.48% | 26.70% |
| G1 to G3a | 0.29% | 1.68% | 3.59% |
| G1 to G3b | 0.08% | 0.48% | 1.03% |
| G1 to G4 | 0.01% | 0.08% | 0.17% |
| G1 to G5 (Pre RRT) | 0.00% | 0.02% | 0.04% |
| G1 to Dialysis | 0.01% | 0.04% | 0.09% |
| G1 to Transplant | 0.01% | 0.04% | 0.09% |
| G2 to G3a | 2.14% | 12.48% | 26.70% |
| G2 to G3b | 0.41% | 2.38% | 5.10% |
| G2 to G4 | 0.07% | 0.44% | 0.93% |
| G2 to G5 (Pre RRT) | 0.00% | 0.02% | 0.04% |
| G2 to Dialysis | 0.01% | 0.04% | 0.09% |
| G2 to Transplant | 0.01% | 0.06% | 0.13% |
| G3a to G3b | 4.24% | 23.40% | 46.27% |
| G3a to G4 | 0.26% | 1.46% | 2.89% |
| G3a to G5 (Pre RRT) | 0.01% | 0.08% | 0.15% |
| G3a to Dialysis | 0.01% | 0.08% | 0.15% |
| G3a to Transplant | 0.02% | 0.10% | 0.19% |
| G3b to G4 | 4.24% | 23.40% | 46.27% |
| G3b to G5 (Pre RRT) | 0.10% | 0.54% | 1.07% |
| G3b to Dialysis | 0.09% | 0.48% | 0.95% |
| G3b to Transplant | 0.05% | 0.25% | 0.50% |
| G4 to G5 (Pre RRT) | 4.24% | 23.40% | 46.27% |
| G4 to Dialysis | 2.57% | 14.78% | 31.11% |
| G4 to Transplant | 0.20% | 1.14% | 2.32% |
| G5 (Pre RRT) to Dialysis | 12.20% | 55.05% | 84.49% |
| G5 to Transplant | 0.96% | 4.35% | 6.67% |
| Dialysis to Transplant | 5.50% | 5.50% | 5.50% |

*G1b or G1c

**G2b or G2c

**Table A3.** Probability of death within each age range*

|  | DKD 1 | DKD 2 | DKD 3** | DKD 4 | DKD 5 | Dialysis | Kidney Transplant |
| --- | --- | --- | --- | --- | --- | --- | --- |
| 0–1 | 0.011 | 0.011 | 0.011 | 0.016 | 0.016 | 0.055 | 0.022 |
| 1–2 | 0.001 | 0.001 | 0.001 | 0.001 | 0.001 | 0.004 | 0.001 |
| 2–3 | 0.000 | 0.000 | 0.000 | 0.001 | 0.001 | 0.002 | 0.001 |
| 3–4 | 0.000 | 0.000 | 0.000 | 0.001 | 0.001 | 0.002 | 0.001 |
| 4–5 | 0.000 | 0.000 | 0.000 | 0.000 | 0.000 | 0.001 | 0.001 |
| 5–6 | 0.000 | 0.000 | 0.000 | 0.000 | 0.000 | 0.001 | 0.001 |
| 6–7 | 0.000 | 0.000 | 0.000 | 0.000 | 0.000 | 0.001 | 0.000 |
| 7–8 | 0.000 | 0.000 | 0.000 | 0.000 | 0.000 | 0.001 | 0.000 |
| 8–9 | 0.000 | 0.000 | 0.000 | 0.000 | 0.000 | 0.001 | 0.000 |
| 9–10 | 0.000 | 0.000 | 0.000 | 0.000 | 0.000 | 0.001 | 0.000 |
| 10–11 | 0.000 | 0.000 | 0.000 | 0.000 | 0.000 | 0.001 | 0.000 |
| 11–12 | 0.000 | 0.000 | 0.000 | 0.000 | 0.000 | 0.001 | 0.000 |
| 12–13 | 0.000 | 0.000 | 0.000 | 0.000 | 0.000 | 0.001 | 0.001 |
| 13–14 | 0.000 | 0.000 | 0.000 | 0.001 | 0.001 | 0.002 | 0.001 |
| 14–15 | 0.001 | 0.001 | 0.000 | 0.001 | 0.001 | 0.003 | 0.001 |
| 15–16 | 0.001 | 0.001 | 0.001 | 0.001 | 0.001 | 0.003 | 0.001 |
| 16–17 | 0.001 | 0.001 | 0.001 | 0.001 | 0.001 | 0.004 | 0.002 |
| 17–18 | 0.001 | 0.001 | 0.001 | 0.001 | 0.001 | 0.005 | 0.002 |
| 18–19 | 0.001 | 0.001 | 0.001 | 0.002 | 0.002 | 0.006 | 0.002 |
| 19–20 | 0.001 | 0.001 | 0.001 | 0.002 | 0.002 | 0.007 | 0.003 |
| 20–21 | 0.002 | 0.002 | 0.001 | 0.002 | 0.002 | 0.008 | 0.003 |
| 21–22 | 0.002 | 0.002 | 0.002 | 0.002 | 0.002 | 0.009 | 0.003 |
| 22–23 | 0.002 | 0.002 | 0.002 | 0.003 | 0.003 | 0.009 | 0.004 |
| 23–24 | 0.002 | 0.002 | 0.002 | 0.003 | 0.003 | 0.010 | 0.004 |
| 24–25 | 0.002 | 0.002 | 0.002 | 0.003 | 0.003 | 0.010 | 0.004 |
| 25–26 | 0.002 | 0.002 | 0.002 | 0.003 | 0.003 | 0.011 | 0.004 |
| 26–27 | 0.002 | 0.002 | 0.002 | 0.003 | 0.003 | 0.011 | 0.004 |
| 27–28 | 0.002 | 0.002 | 0.002 | 0.003 | 0.003 | 0.012 | 0.004 |
| 28–29 | 0.002 | 0.002 | 0.002 | 0.003 | 0.003 | 0.012 | 0.005 |
| 29–30 | 0.003 | 0.003 | 0.002 | 0.004 | 0.004 | 0.013 | 0.005 |
| 30–31 | 0.003 | 0.003 | 0.003 | 0.004 | 0.004 | 0.013 | 0.005 |
| 31–32 | 0.003 | 0.003 | 0.003 | 0.004 | 0.004 | 0.014 | 0.005 |
| 32–33 | 0.003 | 0.003 | 0.003 | 0.004 | 0.004 | 0.015 | 0.006 |
| 33–34 | 0.003 | 0.003 | 0.003 | 0.004 | 0.004 | 0.015 | 0.006 |
| 34–35 | 0.003 | 0.003 | 0.003 | 0.004 | 0.004 | 0.016 | 0.006 |
| 35–36 | 0.003 | 0.003 | 0.003 | 0.005 | 0.005 | 0.017 | 0.006 |
| 36–37 | 0.004 | 0.004 | 0.003 | 0.005 | 0.005 | 0.017 | 0.007 |
| 37–38 | 0.004 | 0.004 | 0.004 | 0.005 | 0.005 | 0.018 | 0.007 |
| 38–39 | 0.004 | 0.004 | 0.004 | 0.005 | 0.005 | 0.019 | 0.007 |
| 39–40 | 0.004 | 0.004 | 0.004 | 0.005 | 0.005 | 0.019 | 0.007 |
| 40–41 | 0.004 | 0.004 | 0.004 | 0.006 | 0.006 | 0.020 | 0.008 |
| 41–42 | 0.004 | 0.004 | 0.004 | 0.006 | 0.006 | 0.021 | 0.008 |
| 42–43 | 0.005 | 0.005 | 0.004 | 0.006 | 0.006 | 0.022 | 0.009 |
| 43–44 | 0.005 | 0.005 | 0.005 | 0.007 | 0.007 | 0.024 | 0.009 |
| 44–45 | 0.005 | 0.005 | 0.005 | 0.007 | 0.007 | 0.025 | 0.010 |
| 45–46 | 0.005 | 0.005 | 0.005 | 0.008 | 0.008 | 0.027 | 0.010 |
| 46–47 | 0.006 | 0.006 | 0.006 | 0.008 | 0.008 | 0.029 | 0.011 |
| 47–48 | 0.006 | 0.006 | 0.006 | 0.009 | 0.009 | 0.031 | 0.012 |
| 48–49 | 0.007 | 0.007 | 0.007 | 0.009 | 0.009 | 0.033 | 0.013 |
| 49–50 | 0.007 | 0.007 | 0.007 | 0.010 | 0.010 | 0.036 | 0.014 |
| 50–51 | 0.008 | 0.008 | 0.008 | 0.011 | 0.011 | 0.039 | 0.015 |
| 51–52 | 0.009 | 0.009 | 0.008 | 0.012 | 0.012 | 0.042 | 0.016 |
| 52–53 | 0.009 | 0.009 | 0.009 | 0.013 | 0.013 | 0.046 | 0.018 |
| 53–54 | 0.010 | 0.010 | 0.010 | 0.014 | 0.014 | 0.051 | 0.020 |
| 54–55 | 0.011 | 0.011 | 0.011 | 0.016 | 0.016 | 0.056 | 0.022 |
| 55–56 | 0.013 | 0.013 | 0.012 | 0.017 | 0.017 | 0.061 | 0.024 |
| 56–57 | 0.014 | 0.014 | 0.013 | 0.019 | 0.019 | 0.066 | 0.026 |
| 57–58 | 0.015 | 0.015 | 0.014 | 0.020 | 0.020 | 0.071 | 0.028 |
| 58–59 | 0.016 | 0.016 | 0.015 | 0.022 | 0.022 | 0.076 | 0.030 |
| 59–60 | 0.017 | 0.017 | 0.016 | 0.024 | 0.024 | 0.082 | 0.032 |
| 60–61 | 0.019 | 0.019 | 0.018 | 0.025 | 0.025 | 0.089 | 0.035 |
| 61–62 | 0.020 | 0.020 | 0.019 | 0.027 | 0.027 | 0.096 | 0.038 |
| 62–63 | 0.021 | 0.021 | 0.021 | 0.029 | 0.029 | 0.102 | 0.040 |
| 63–64 | 0.023 | 0.023 | 0.022 | 0.031 | 0.031 | 0.109 | 0.043 |
| 64–65 | 0.024 | 0.024 | 0.023 | 0.033 | 0.033 | 0.115 | 0.046 |
| 65–66 | 0.026 | 0.026 | 0.025 | 0.035 | 0.035 | 0.122 | 0.048 |
| 66–67 | 0.027 | 0.027 | 0.026 | 0.038 | 0.038 | 0.129 | 0.052 |
| 67–68 | 0.029 | 0.029 | 0.028 | 0.040 | 0.040 | 0.138 | 0.055 |
| 68–69 | 0.032 | 0.032 | 0.030 | 0.043 | 0.043 | 0.148 | 0.059 |
| 69–70 | 0.034 | 0.034 | 0.033 | 0.047 | 0.047 | 0.159 | 0.064 |
| 70–71 | 0.037 | 0.037 | 0.035 | 0.050 | 0.050 | 0.170 | 0.069 |
| 71–72 | 0.040 | 0.040 | 0.038 | 0.055 | 0.055 | 0.184 | 0.075 |
| 72–73 | 0.043 | 0.043 | 0.041 | 0.059 | 0.059 | 0.196 | 0.080 |
| 73–74 | 0.048 | 0.048 | 0.046 | 0.065 | 0.065 | 0.216 | 0.089 |
| 74–75 | 0.052 | 0.052 | 0.050 | 0.071 | 0.071 | 0.232 | 0.096 |
| 75–76 | 0.057 | 0.057 | 0.055 | 0.078 | 0.078 | 0.254 | 0.106 |
| 76–77 | 0.063 | 0.063 | 0.060 | 0.085 | 0.085 | 0.275 | 0.116 |
| 77–78 | 0.069 | 0.069 | 0.067 | 0.094 | 0.094 | 0.301 | 0.128 |
| 78–79 | 0.077 | 0.077 | 0.074 | 0.104 | 0.104 | 0.327 | 0.140 |
| 79–80 | 0.085 | 0.085 | 0.081 | 0.114 | 0.114 | 0.356 | 0.155 |
| 80–81 | 0.093 | 0.093 | 0.090 | 0.126 | 0.126 | 0.385 | 0.170 |
| 81–82 | 0.103 | 0.103 | 0.099 | 0.139 | 0.139 | 0.417 | 0.187 |
| 82–83 | 0.115 | 0.115 | 0.110 | 0.154 | 0.154 | 0.455 | 0.207 |
| 83–84 | 0.128 | 0.128 | 0.123 | 0.172 | 0.172 | 0.494 | 0.229 |
| 84–85 | 0.141 | 0.141 | 0.136 | 0.189 | 0.189 | 0.532 | 0.252 |
| 85–86 | 0.158 | 0.158 | 0.152 | 0.211 | 0.211 | 0.575 | 0.279 |
| 86–87 | 0.175 | 0.175 | 0.169 | 0.233 | 0.233 | 0.617 | 0.307 |
| 87–88 | 0.195 | 0.195 | 0.188 | 0.258 | 0.258 | 0.660 | 0.338 |
| 88–89 | 0.217 | 0.217 | 0.209 | 0.285 | 0.285 | 0.703 | 0.371 |
| 89–90 | 0.240 | 0.240 | 0.231 | 0.314 | 0.314 | 0.744 | 0.406 |
| 90–91 | 0.264 | 0.264 | 0.255 | 0.345 | 0.345 | 0.783 | 0.442 |
| 91–92 | 0.291 | 0.291 | 0.281 | 0.376 | 0.376 | 0.819 | 0.479 |
| 92–93 | 0.318 | 0.318 | 0.307 | 0.409 | 0.409 | 0.851 | 0.517 |
| 93–94 | 0.347 | 0.347 | 0.335 | 0.443 | 0.443 | 0.880 | 0.555 |
| 94–95 | 0.377 | 0.377 | 0.364 | 0.478 | 0.478 | 0.905 | 0.593 |
| 95–96 | 0.407 | 0.407 | 0.394 | 0.513 | 0.513 | 0.926 | 0.630 |
| 96–97 | 0.438 | 0.438 | 0.425 | 0.547 | 0.547 | 0.943 | 0.666 |
| 97–98 | 0.469 | 0.469 | 0.455 | 0.582 | 0.582 | 0.957 | 0.700 |
| 98–99 | 0.500 | 0.500 | 0.486 | 0.615 | 0.615 | 0.968 | 0.733 |
| 99–100 | 0.531 | 0.531 | 0.516 | 0.647 | 0.647 | 0.977 | 0.763 |
| 100+ | 1.000 | 1.000 | 1.000 | 1.000 | 1.000 | 1.000 | 1.000 |
| *SMRs: DKD 1 = 1.05; DKD 2 = 1.05; DKD 3 = 0.97; DKD 4 = 1.82; DKD 5 = 1.82; Dialysis = 10.20; Kidney Transplant = 3.90  **Utilized for stages 3a and 3b in the model | | | | | | | |

**Table A4.** CV Events with MRAs (Scenario 2)

| Disease State | CV Event Rate | | |
| --- | --- | --- | --- |
|  | Low Risk | Intermediate Risk | High Risk |
| DKD Stage 1 | | | |
| KidneyIntelX | 4.18% | 3.87% | 3.47% |
| KDIGO | 4.18% | 3.99% | 3.98% |
| DKD Stage 2 | | | |
| KidneyIntelX | 4.18% | 3.87% | 3.47% |
| KDIGO | 4.18% | 3.99% | 3.97% |
| DKD Stage 3a | | | |
| KidneyIntelX | 5.11% | 4.73% | 4.24% |
| KDIGO | 5.11% | 4.91% | 4.90% |
| DKD Stage 3b | | | |
| KidneyIntelX | 5.84% | 5.41% | 4.85% |
| KDIGO | 5.84% | 5.61% | 5.60% |
| DKD Stage 4 | | | |
| KidneyIntelX | 12.21% | 12.02% | 12.00% |
| KDIGO | 12.21% | 12.02% | 12.00% |
| DKD Stage 5 | | | |
| KidneyIntelX | 12.21% | 12.03% | 12.00% |
| KDIGO | 12.21% | 12.03% | 12.02% |

**Table A5.** Office visit costs & probabilities

| Drug Type | Cost ($) | Percent of Patients Referred (%) | | | | | |  |
| --- | --- | --- | --- | --- | --- | --- | --- | --- |
|  |  | DKD 1 | DKD 2 | DKD 3a | DKD 3b | DKD 4 | DKD 5 |  |
| *Low Risk* | | | | | | | | |
| *Nephrologist Office Visit (1st Time)* | $233.70 |  |  |  |  |  |  |  |
| KidneyIntelX |  | 0.40% | 0.80% | 8.70% | 8.70% | 8.70% | 8.70% |  |
| KDIGO |  | 0.40% | 0.80% | 8.70% | 8.70% | 8.70% | 8.70% |  |
| *Nephrologist Office Visit (Follow Up)* | $159.85 |  |  |  |  |  |  |  |
| KidneyIntelX |  | 0.40% | 0.80% | 8.70% | 8.70% | 8.70% | 8.70% |  |
| KDIGO |  | 0.40% | 0.80% | 8.70% | 8.70% | 8.70% | 8.70% |  |
| *Dietician (1st Time)* | $44.44 |  |  |  |  |  |  |  |
| KidneyIntelX |  | 0.40% | 0.80% | 8.70% | 8.70% | 8.70% | 8.70% |  |
| KDIGO |  | 0.40% | 0.80% | 8.70% | 8.70% | 8.70% | 8.70% |  |
| *Dietician (Follow Up)* | $38.25 |  |  |  |  |  |  |  |
| KidneyIntelX |  | 0.40% | 0.80% | 8.70% | 8.70% | 8.70% | 8.70% |  |
| KDIGO |  | 0.40% | 0.80% | 8.70% | 8.70% | 8.70% | 8.70% |  |
| *CKD Education Services* | $137.96 |  |  |  |  |  |  |  |
| KidneyIntelX |  | 0.40% | 0.80% | 8.70% | 8.70% | 8.70% | 8.70% |  |
| KDIGO |  | 0.40% | 0.80% | 8.70% | 8.70% | 8.70% | 8.70% |  |
| *Social Worker (Behavioral Assessment)* | $27.42 |  |  |  |  |  |  |  |
| KidneyIntelX |  | 0.40% | 0.80% | 8.70% | 8.70% | 8.70% | 8.70% |  |
| KDIGO |  | 0.40% | 0.80% | 8.70% | 8.70% | 8.70% | 8.70% |  |
| *Behavioral Re-Assessment* | $27.42 |  |  |  |  |  |  |  |
| KidneyIntelX |  | 0.40% | 0.80% | 8.70% | 8.70% | 8.70% | 8.70% |  |
| KDIGO |  | 0.40% | 0.80% | 8.70% | 8.70% | 8.70% | 8.70% |  |
| *Intermediate Risk* | | | | | | | | |
| *Nephrologist Office Visit (1st Time)* | $233.70 |  |  |  |  |  |  |  |
| KidneyIntelX |  | 0.40% | 0.80% | 8.70% | 8.70% | 8.70% | 8.70% |  |
| KDIGO |  | 0.40% | 0.80% | 8.70% | 8.70% | 8.70% | 8.70% |  |
| *Nephrologist Office Visit (Follow Up)* | $159.85 |  |  |  |  |  |  |  |
| KidneyIntelX |  | 0.40% | 0.80% | 8.70% | 8.70% | 8.70% | 8.70% |  |
| KDIGO |  | 0.40% | 0.80% | 8.70% | 8.70% | 8.70% | 8.70% |  |
| *Dietician (1st Time)* | $44.44 |  |  |  |  |  |  |  |
| KidneyIntelX |  | 0.40% | 0.80% | 8.70% | 8.70% | 8.70% | 8.70% |  |
| KDIGO |  | 0.40% | 0.80% | 8.70% | 8.70% | 8.70% | 8.70% |  |
| *Dietician (Follow Up)* | $38.25 |  |  |  |  |  |  |  |
| KidneyIntelX |  | 0.40% | 0.80% | 8.70% | 8.70% | 8.70% | 8.70% |  |
| KDIGO |  | 0.40% | 0.80% | 8.70% | 8.70% | 8.70% | 8.70% |  |
| *CKD Education Services* | $137.96 |  |  |  |  |  |  |  |
| KidneyIntelX |  | 0.40% | 0.80% | 8.70% | 8.70% | 8.70% | 8.70% |  |
| KDIGO |  | 0.40% | 0.80% | 8.70% | 8.70% | 8.70% | 8.70% |  |
| *Social Worker (Behavioral Assessment)* | $27.42 |  |  |  |  |  |  |  |
| KidneyIntelX |  | 0.40% | 0.80% | 8.70% | 8.70% | 8.70% | 8.70% |  |
| KDIGO |  | 0.40% | 0.80% | 8.70% | 8.70% | 8.70% | 8.70% |  |
| *Behavioral Re-Assessment* | $27.42 |  |  |  |  |  |  |  |
| KidneyIntelX |  | 0.40% | 0.80% | 8.70% | 8.70% | 8.70% | 8.70% |  |
| KDIGO |  | 0.40% | 0.80% | 8.70% | 8.70% | 8.70% | 8.70% |  |
| *High Risk* | | | | | | | | |
| *Nephrologist Office Visit (1st Time)* | $233.70 |  |  |  |  |  |  |  |
| KidneyIntelX |  | 15.00% | 15.00% | 25.00% | 30.00% | 30.00% | 30.00% |  |
| KDIGO |  | 0.40% | 0.80% | 8.70% | 8.70% | 8.70% | 8.70% |  |
| *Nephrologist Office Visit (Follow Up)* | $159.85 |  |  |  |  |  |  |  |
| KidneyIntelX |  | 15.00% | 15.00% | 25.00% | 30.00% | 30.00% | 30.00% |  |
| KDIGO |  | 0.40% | 0.80% | 8.70% | 8.70% | 8.70% | 8.70% |  |
| *Dietician (1st Time)* | $44.44 |  |  |  |  |  |  |  |
| KidneyIntelX |  | 10.00% | 10.00% | 20.00% | 25.00% | 25.00% | 25.00% |  |
| KDIGO |  | 0.40% | 0.80% | 8.70% | 8.70% | 8.70% | 8.70% |  |
| *Dietician (Follow Up)* | $38.25 |  |  |  |  |  |  |  |
| KidneyIntelX |  | 10.00% | 10.00% | 20.00% | 25.00% | 25.00% | 25.00% |  |
| KDIGO |  | 0.40% | 0.80% | 8.70% | 8.70% | 8.70% | 8.70% |  |
| *CKD Education Services* | $137.96 |  |  |  |  |  |  |  |
| KidneyIntelX |  | 10.00% | 10.00% | 20.00% | 25.00% | 25.00% | 25.00% |  |
| KDIGO |  | 0.40% | 0.80% | 8.70% | 8.70% | 8.70% | 8.70% |  |
| *Social Worker (Behavioral Assessment)* | $27.42 |  |  |  |  |  |  |  |
| KidneyIntelX |  | 10.00% | 10.00% | 20.00% | 25.00% | 25.00% | 25.00% |  |
| KDIGO |  | 0.40% | 0.80% | 8.70% | 8.70% | 8.70% | 8.70% |  |
| *Behavioral Re-Assessment* | $27.42 |  |  |  |  |  |  |  |
| KidneyIntelX |  | 10.00% | 10.00% | 20.00% | 25.00% | 25.00% | 25.00% |  |
| KDIGO |  | 0.40% | 0.80% | 8.70% | 8.70% | 8.70% | 8.70% |  |

**Table A6.** Medication Costs & Prescription Rates

| Drug Type | Monthly  Cost ($)* | Percent of Patients Receiving (%) | | | | | |  |
| --- | --- | --- | --- | --- | --- | --- | --- | --- |
|  |  | DKD 1 | DKD 2 | DKD 3a | DKD 3b | DKD 4 | DKD 5 | |
| *Low Risk* | | | | | | | |  |
| *ACE Inhibitor/ARB* | $69.21 |  |  |  |  |  |  | |
| KidneyIntelX |  | 81% | 81% | 81% | 81% | 81% | 81% | |
| KDIGO |  | 81% | 81% | 81% | 81% | 81% | 81% | |
| *Diuretic* | $19.15 |  |  |  |  |  |  | |
| KidneyIntelX |  | 40% | 45% | 45% | 45% | 45% | 45% | |
| KDIGO |  | 40% | 45% | 45% | 45% | 45% | 45% | |
| *Calcium Channel Blocker* | $55.26 |  |  |  |  |  |  | |
| KidneyIntelX |  | 0% | 5% | 5% | 5% | 5% | 5% | |
| KDIGO |  | 0% | 5% | 5% | 5% | 5% | 5% | |
| *Sulfonylureas (Glipizide)* | $21.11 |  |  |  |  |  |  | |
| KidneyIntelX |  | 20% | 25% | 30% | 25% | 25% | 25% | |
| KDIGO |  | 20% | 25% | 30% | 25% | 25% | 25% | |
| *Biguanides* | $49.24 |  |  |  |  |  |  | |
| KidneyIntelX |  | 20% | 30% | 40% | 45% | 45% | 45% | |
| KDIGO |  | 20% | 30% | 40% | 45% | 45% | 45% | |
| *SGLT2 (Dapagliflozin)* | $666.46 |  |  |  |  |  |  | |
| KidneyIntelX |  | 4% | 4% | 4% | 4% | 4% | 4% | |
| KDIGO |  | 11% | 11% | 9% | 9% | 4% | 4% | |
| *MRAs (Finerenone)*** | $606.36 |  |  |  |  |  |  | |
| KidneyIntelX |  | 1% | 1% | 1% | 1% | 0% | 0% | |
| KDIGO |  | 1% | 1% | 1% | 1% | 0% | 0% | |
| *Intermediate Risk* | | | | | | | |  |
| *ACE Inhibitor/ARB* | $69.21 |  |  |  |  |  |  | |
| KidneyIntelX |  | 81% | 81% | 81% | 81% | 81% | 81% | |
| KDIGO |  | 81% | 81% | 81% | 81% | 81% | 81% | |
| *Diuretic* | $19.15 |  |  |  |  |  |  | |
| KidneyIntelX |  | 40% | 45% | 45% | 45% | 45% | 45% | |
| KDIGO |  | 40% | 45% | 45% | 45% | 45% | 45% | |
| *Calcium Channel Blocker* | $55.26 |  |  |  |  |  |  | |
| KidneyIntelX |  | 0% | 5% | 5% | 5% | 5% | 5% | |
| KDIGO |  | 0% | 5% | 5% | 5% | 5% | 5% | |
| *Sulfonylureas (Glipizide)* | $21.11 |  |  |  |  |  |  | |
| KidneyIntelX |  | 20% | 25% | 30% | 25% | 25% | 25% | |
| KDIGO |  | 20% | 25% | 30% | 25% | 25% | 25% | |
| *Biguanides* | $49.24 |  |  |  |  |  |  | |
| KidneyIntelX |  | 20% | 30% | 40% | 45% | 45% | 45% | |
| KDIGO |  | 20% | 30% | 40% | 45% | 45% | 45% | |
| *SGLT2i (Dapagliflozin)* | $666.46 |  |  |  |  |  |  | |
| KidneyIntelX |  | 16% | 16% | 16% | 16% | 4% | 4% | |
| KDIGO |  | 11% | 11% | 9% | 9% | 4% | 4% | |
| *MRAs (Finerenone)*** | $606.36 |  |  |  |  |  |  | |
| KidneyIntelX |  | 3% | 3% | 3% | 3% | 0% | 0% | |
| KDIGO |  | 1% | 1% | 1% | 1% | 0% | 0% | |
| *High Risk* | | | | | | | | |
| *ACE Inhibitor/ARBs* | $69.21 |  |  |  |  |  |  | |
| KidneyIntelX |  | 81% | 81% | 81% | 81% | 81% | 81% | |
| KDIGO |  | 81% | 81% | 81% | 81% | 81% | 81% | |
| *Diuretic* | $19.15 |  |  |  |  |  |  | |
| KidneyIntelX |  | 35% | 30% | 20% | 15% | 15% | 15% | |
| KDIGO |  | 40% | 45% | 45% | 45% | 45% | 45% | |
| *Calcium Channel Blocker* | $55.26 |  |  |  |  |  |  | |
| KidneyIntelX |  | 5% | 5% | 5% | 5% | 5% | 5% | |
| KDIGO |  | 5% | 5% | 5% | 5% | 5% | 5% | |
| *Sulfonylureas (Glipizide)* | $21.11 |  |  |  |  |  |  | |
| KidneyIntelX |  | 5% | 10% | 10% | 5% | 5% | 5% | |
| KDIGO |  | 20% | 25% | 30% | 25% | 25% | 25% | |
| *Biguanides* | $49.24 |  |  |  |  |  |  | |
| KidneyIntelX |  | 20% | 25% | 30% | 35% | 35% | 35% | |
| KDIGO |  | 20% | 30% | 40% | 45% | 45% | 45% | |
| *SGLT2 (Dapagliflozin)* | $666.46 |  |  |  |  |  |  | |
| KidneyIntelX |  | 37% | 37% | 37% | 37% | 4% | 4% | |
| KDIGO |  | 11% | 11% | 9% | 9% | 4% | 4% | |
| *MRAs (Finerenone)*** | $606.36 |  |  |  |  |  |  | |
| KidneyIntelX |  | 7% | 7% | 7% | 7% | 0% | 0% | |
| KDIGO |  | 1% | 1% | 1% | 1% | 0% | 0% | |

*2024 USD
**Scenario 2 only; 0% in the base case

**Figure A1.** KDIGO Heat Map [63]
